# Optical control of neuronal activity using a light-operated GIRK channel opener (LOGO)†

**DOI:** 10.1039/c5sc04084a

**Published:** 2015-12-23

**Authors:** David M. Barber, Matthias Schönberger, Jessica Burgstaller, Joshua Levitz, C. David Weaver, Ehud Y. Isacoff, Herwig Baier, Dirk Trauner

**Affiliations:** a Department of Chemistry and Center for Integrated Protein Science, Ludwig Maximilians University Munich Butenandtstraße 5-13 81377 Munich Germany dirk.trauner@lmu.de; b Max Planck Institute of Neurobiology Am Klopferspitz 18 82152 Martinsried Germany; c Department of Molecular and Cell Biology and Helen Wills Neuroscience Institute, University of California Berkeley California USA; d Department of Pharmacology and Institute of Chemical Biology, Vanderbilt University School of Medicine Nashville Tennessee 37232 USA; e Physical Bioscience Division, Lawrence Berkeley National Laboratory Berkeley California USA

## Abstract

G-protein coupled inwardly rectifying potassium (GIRK) channels are expressed throughout the human body and are an integral part of inhibitory signal transduction pathways. Upon binding of G_βγ_ subunits released from G-protein coupled receptors (GPCRs), GIRK channels open and reduce the activity of excitable cells *via* hyperpolarization. As such, they play a role in cardiac output, the coordination of movement and cognition. Due to their involvement in a multitude of pathways, the precision control of GIRK channels is an important endeavour. Here, we describe the development of the photoswitchable agonist LOGO (the Light-Operated GIRK channel Opener), which activates GIRK channels in the dark and is rapidly deactivated upon exposure to long wavelength UV irradiation. LOGO is the first photochromic K^+^ channel opener and selectively targets channels that contain the GIRK1 subunit. It can be used to optically silence action potential firing in dissociated hippocampal neurons and LOGO exhibits activity *in vivo*, controlling the motility of zebrafish larvae in a light-dependent fashion. We envisage that LOGO will be a valuable research tool to dissect the function of GIRK channels from other GPCR dependent signalling pathways.

## Abstract 2

We describe the development of the photoswitchable agonist LOGO, which activates GIRK channels in the dark and is rapidly deactivated upon exposure to long wavelength UV irradiation. LOGO can be used to optically silence action potential firing in dissociated hippocampal neurons and exhibits activity *in vivo*, controlling the motility of zebrafish larvae in a light-dependent fashion.

## Graphical abstract

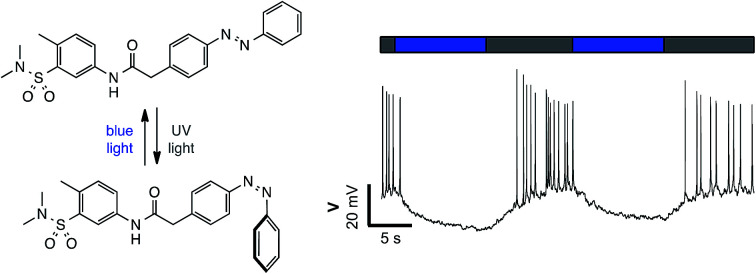

## Introduction

G-protein coupled inwardly rectifying potassium (GIRK) channels are an integral link between metabotropic and ionotropic pathways in neurons. They constitute a subclass of inwardly rectifying potassium channels that are natively activated by G-protein coupled receptors (GPCRs) through interactions with G_βγ_ subunits.^1^ One of the most important effects of GIRK activation and subsequent channel opening is the hyperpolarization of cell membranes, which immediately results in a reduction of activity in excitable cells. Therefore, neurotransmitters that target both ion channels and GPCRs, including glutamate, γ-aminobutyric acid, acetylcholine, dopamine and serotonin, can have inhibitory affects. As a consequence, GIRK channels play crucial roles in cognition, nociception, coordination, energy homeostasis and cardiac output.^2^ Therefore, it is unsurprising that the malfunction of GIRK channels is associated with several neurological and cardiological disorders.^3^

GIRK channels are comprised of homo- or heterotetramers formed by the four subunits GIRK1-4.^4^ Although the expression patterns of GIRK subunits differs throughout the body, the prototypical GIRK channels of the central nervous system (CNS) are GIRK1/2 heteromers and the isoforms found in the cardiovascular system are GIRK1/4 channels.^5^ Recent breakthroughs in structural biology have identified the molecular basis for GIRK channel function, such as inward rectification and polyamine block, as well as intracellular activation by sodium ions and lipids.^6^

**Fig. 1:**
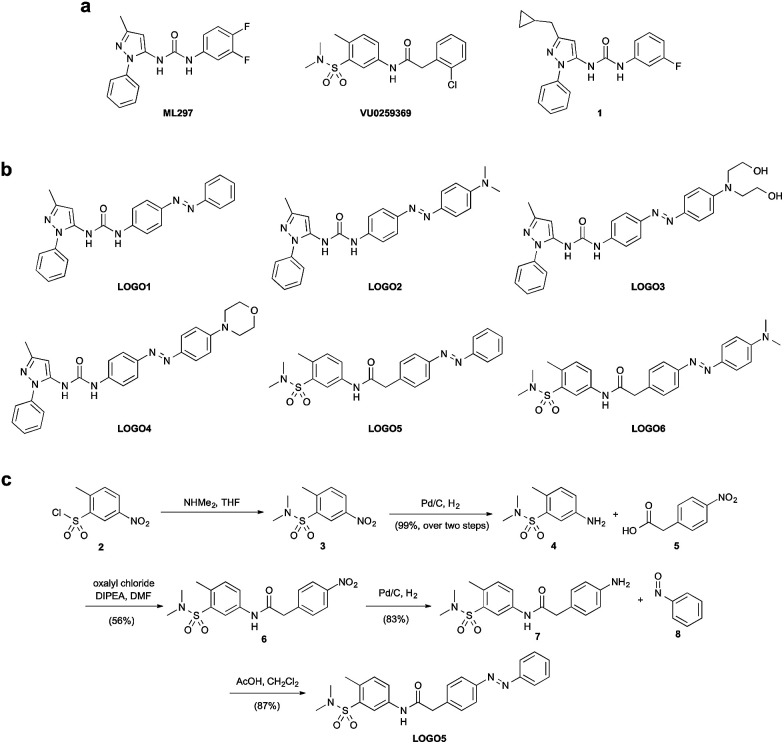
Design of the photoswitchable GIRK channel agonists LOGO1–6 using an ‘azo-extension’ strategy. (a) Chemical structures of the GIRK channel agonists ML297 and VU0259369, and the GIRK channel inhibitor 1. (b) Chemical structures of the photoswitchable GIRK agonists LOGO1–6. (c) Synthesis of LOGO5 using a Mills reaction.

It has been demonstrated that GIRK channels are inhibited by a variety of drugs, as well as polyamines and barium ions.^7^ However, these compounds are not selective for GIRK channels.^8,9^ GIRK channels can also be activated by small molecules including volatile anesthetics,^10^ ethanol^11^ and the natural product naringin.^12^ Again these compounds either exhibit poor selectivity, poor potency or a combination of the two. However, the landscape of GIRK channel research was recently transformed by two small molecules, ML297 and VU0259369 (Fig. 1a).^13^ These compounds were shown to be the first potent and selective activators of GIRK channels bearing the GIRK1 subunit. Subtle changes to the molecular structure of ML297 resulted in the creation of potent GIRK1 channel inhibitors, an example of which is compound 1 (Fig. 1a).^14^

With the first small molecular agonists of GIRK channels at our disposal, we sought to develop photoswitchable versions that can endow light control to GIRK channels.^15^ This approach, termed photopharmacology, is able to confer exquisite spatiotemporal control onto biological systems using the unrivalled precision of light and has previously been used to optically control ion channels,^16^ GPCRs,^17^ enzymes^18^ and antibiotics.^19^

Herein, we present LOGO (the Light-Operated GIRK channel Opener), an azobenzene photoswitch based on VU0259369 that endows GIRK channels with remarkable photocontrol using UV and blue light. LOGO is the first example of a photochromic K^+^ channel opener, as opposed to blocker,^20^ and the first photoswitch to selectively target GIRK channels. We envisage that LOGO will be a valuable tool for GIRK channel research, allowing the dissection of GIRK signalling from other GPCR-dependent pathways. This will further our understanding of GIRK channel function and facilitate their development as a therapeutic target.

## Results and discussion

Using the compounds ML297 and VU0259369 as a basis for our photoswitch design, we employed an ‘azo-extension’ strategy to furnish our family of LOGOs (Fig. 1b).^21^ In the ML297 series, we prepared LOGO1–4 bearing different electron-donating groups in the 4′-position of the azobenzene. In a similar fashion, LOGO5 and LOGO6 were derived from the compound VU0259369. The representative synthesis of LOGO5 started from sulfonyl chloride 2. Nucleophilic substitution with dimethylamine, followed by reduction of the aromatic nitro group, furnished sulfonamide 4 (Fig. 1c). An amide coupling reaction between 4 and 4-nitrophenylacetic acid (5) afforded nitro amide 6, which was then reduced to the aniline 7. Mills reaction of aniline 7 with nitrosobenzene (8) in the presence of acetic acid then afforded LOGO5. The synthesis of LOGO1–4 and LOGO6 proceeded in a similar fashion and is discussed in the ESI (Fig. S1†).

**Fig. 2:**
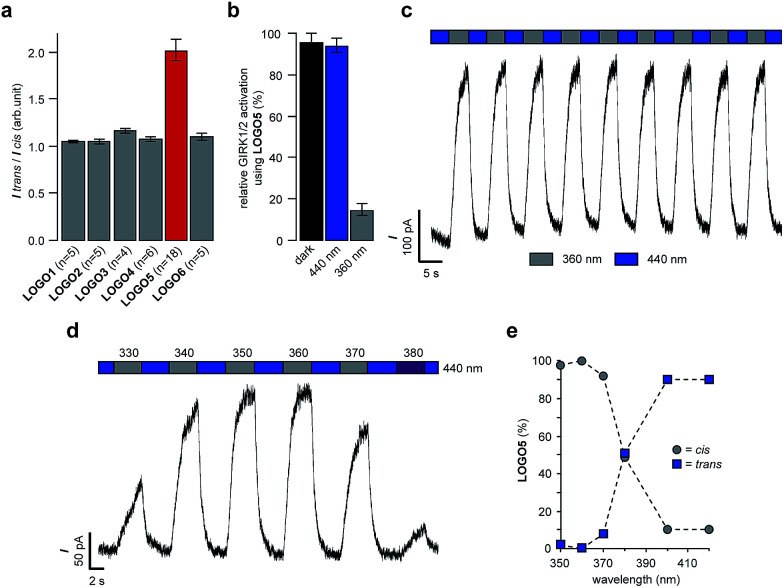
Whole-cell patch clamp electrophysiology of HEK293T cells expressing GIRK1/2 channels. (a) Evaluation of LOGO1–6 (1 μm) revealed that LOGO5 displayed the best photoswitching behaviour. (b) LOGO5 (10 μm) is almost as efficacious as VU0259369 (10 μm) in the dark and under blue light (440 nm) illumination (*n* = 4 cells). (c) The photoswitching of LOGO5 was shown to be reversible over many switching cycles in voltage-clamp mode. (d) Action spectrum of LOGO5 (1 μm) showing the accurate control of cellular currents by toggling the illumination wavelength between 440 nm and 330–380 nm. (e) Photoswitching of LOGO5 in DMSO-d_6_ solution using ^1^H NMR spectroscopy (see ESI for details†). Values represent mean ± SEM.

With our small library of LOGOs in hand, we set about determining their photoswitching properties using UV-Vis spectroscopy. From these experiments, we found the optimum wavelengths for the photoswitching of each of the compounds (Fig. S2†). This information was then applied to our initial compound evaluation using patch clamp electrophysiology in HEK293T cells expressing GIRK1/2 channels (Fig. 2a). Beginning with LOGO1, we found that it could efficiently open GIRK1/2 channels as its *trans*-isomer. However, upon isomerisation to its *cis*-isomer using UV light (390 nm), only a small decrease of the current in the patch clamp experiment was observed. Based on these results, we postulated that LOGO2–4 would endow the GIRK channels with more light-dependent current because of the increased steric interactions between the ligand-binding site of the GIRK channel and the substituents in the 4′-position of the azobenzene. Although a small increase in the photoswitching of the GIRK channel was observed, it was still minor compared to the overall activation of the channel. We therefore turned our attention to LOGO5 and LOGO6, which were derived from VU0259369 instead of ML297. Pleasingly, we discovered that LOGO5 not only activates GIRK1/2 channels, but is an excellent photoswitch (Fig. 2a). We next evaluated the red-shifted photoswitch LOGO6, but to our surprise the photocurrent achieved was drastically reduced compared to that of LOGO5 (Fig. 2a). Therefore, our future investigations and biological evaluation focused on LOGO5. Overall, analysis of LOGO1–6 demonstrated that potent and efficacious ligands could be easily obtained by extending portions of known agonists (Fig. 1a) to include azobenzenes. By contrast, there is a narrow ‘structural window’ for photoswitchable agonists, that is compounds that dramatically change their efficacy upon light-isomerisation.

Having determined that LOGO5 is our optimal photoswitch for the control of GIRK1/2 channels, we investigated its properties in detail using patch clamp electrophysiology and NMR spectroscopy. Firstly, we established that in the dark LOGO5 (10 μm) is almost as efficacious as VU0259369 (10 μm). The same is true under blue light illumination, where *trans*-LOGO5 also predominates (Fig. 2b). Photoisomerisation using UV light (360 nm) then showed that *cis*-LOGO5 is considerably less active on GIRK1/2 channels (Fig. 2b and S3†). Reversible photoactivation with LOGO5 is very robust, with no noticeable loss of photocurrent over several switching cycles in voltage-clamp mode (Fig. 2c). Highly reproducible photoswitching of LOGO5 was also obtained when operating in current-clamp mode (Fig. S4†). Next we determined the action spectrum of LOGO5. When the wavelength was switched between blue light (440 nm) and different wavelengths of UV light (330–380 nm), we observed large differences in current (Fig. 2d). The minimal inward current was observed at 360 nm, whereas illumination at 330, 340, 350, 370 and 380 nm gave more inward current. This corresponds to the photostationary states (PSS) of LOGO5 in DMSO-d_6_ solution as determined by ^1^H NMR spectroscopy (Fig. 2e). Here 100% *cis*-LOGO5 content was observed at 360 nm, whereas 370 and 380 nm *etc.* gave increasing *trans*-LOGO5 content. Hence the concentration of active *trans*-LOGO5 can be controlled with the colour of the light (photodosing).^22^ The kinetics of LOGO5 activation were shown to be fast, with illumination at 440 nm for 1 second resulting in almost complete opening of the GIRK1/2 channel (Fig. S6†).

**Fig. 3:**
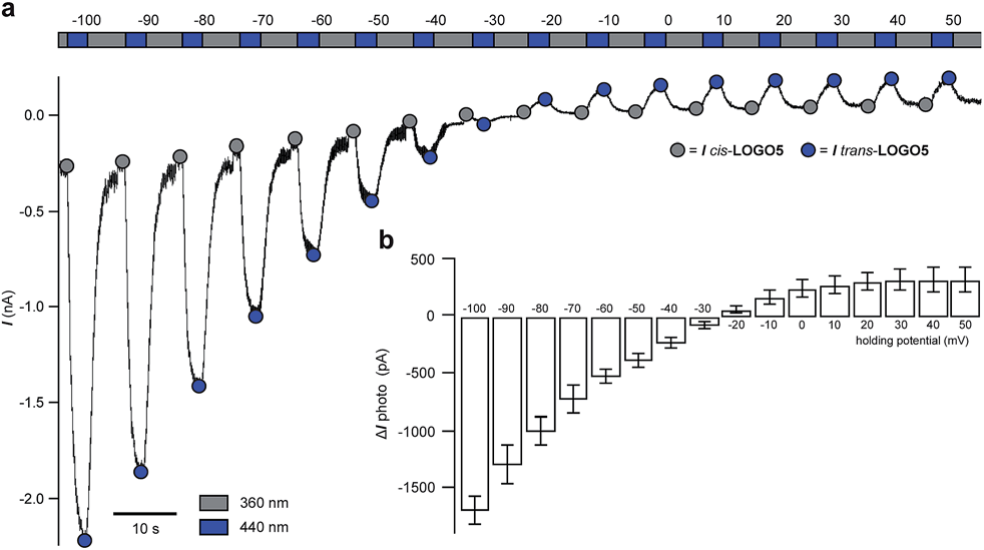
Inward rectification of GIRK1/2 channels in the presence of LOGO5 (10 μm) in voltage-clamp mode. (a) The holding potential was elevated in 10 mV steps from −100 mV to +50 mV illuminating with 360/440/360 nm at each step to give the *cis*-isomer of LOGO5 (360 nm) and the *trans*-isomer of LOGO5 (440 nm). Respective peak currents are indicated as grey and blue circles. (b) Averaged photocurrent amplitudes (Δ*I* = *I*_*trans*-LOGO5_ – *I*_*cis*-LOGO5_). Inward rectification is also translated into the photocurrents Δ*I* (*n* = 3 cells). Values represent mean ± SEM.

Next, we monitored the action of LOGO5 on GIRK1/2 channels expressed in HEK293T cells by elevating the holding potential in 10 mV steps from −100 mV to +50 mV. During each voltage step illumination was switched between 360/440/360 nm (Fig. 3a). Again, we found that the *cis*-isomer of LOGO5 has very little activity at GIRK1/2 channels, whilst the *trans*-isomer of LOGO5 shows strong GIRK1/2 activation. The amplitude of photocurrents at different holding potentials reflects the inward rectification of GIRK1/2 channels (Fig. 3b). Given the high concentration of K^+^ ions (50 mm), the reversal potential was found to lie between −30 and −20 mV.

**Fig. 4:**
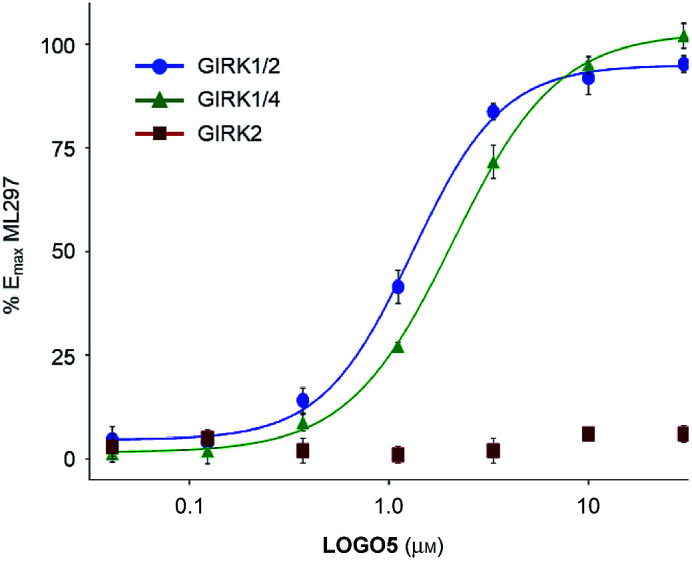
Potency, efficacy and selectivity of LOGO5. Shown are fits to representative data obtained from testing multiple concentrations of LOGO5 on cell lines stably expressing GIRK1/2 (blue circles), GIRK1/4 (green triangles) and GIRK2 (red squares). The measured potencies for GIRK1/2 and GIRK1/4 were 1.2 ± 0.09 μm and 1.9 ± 0.10 μm, respectively. LOGO5 was inactive on cells that only express GIRK2. Efficacy values were normalised to the maximum activity observed using ML297 (10 μm) on GIRK1/2-expressing cells. Error bars represent ± SEM obtained from triplicate wells. Reported potency values represent the SEM obtained from three independent experiments.

To evaluate the activity of LOGO5 at different GIRK channel subtypes, we next employed the thallium flux assay technique (Fig. 4).^23^ We found that LOGO5 is capable of activating GIRK channels that contain the GIRK1 subunit with similar potency and efficacy (GIRK1/2: EC_50_ = 1.2 ± 0.09 μm, % *E*_max_ = 95 ± 5.0; GIRK1/4: EC_50_ = 1.9 ± 0.10 μm, % *E*_max_ = 101.5 ± 6.4; Table S1†). However, LOGO5 is unable to activate homodimeric GIRK2 channels, even at high concentrations.

**Fig. 5:**
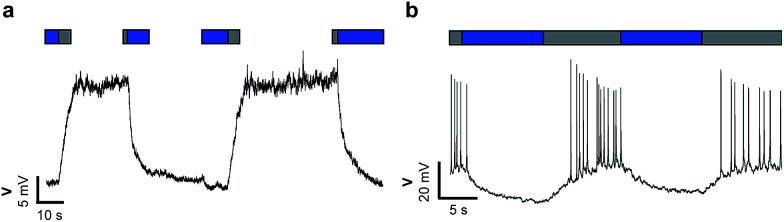
Optical control of excitability *via* endogenous GIRK channels using LOGO5 in rat hippocampal neurons. (a) Photoswitching of LOGO5 reversibly and repeatedly manipulated membrane potentials by 10–20 mV. Light responses were stable in the dark for tens of seconds due to the slow relaxation of the *cis*- and *trans*-isomers of LOGO5. (b) Photoswitching of LOGO5 results in the control of spontaneous action potential firing. (grey = 360 nm; blue = 450 nm).

Having demonstrated LOGO5 on HEK293T cells, which heterologously express GIRK1/2 channels, we next wondered if this tool could be used to control excitable cells that natively express GIRK channels. To test this, we turned to dissociated rat hippocampal neurons which have been shown to express a variety of GIRK subunits.^24^ After the application of LOGO5 (20 μm), pyramidal neurons exhibited large membrane hyperpolarization (15.8 ± 2.5 mV, *n* = 7 cells) in response to illumination with blue light (450 nm), which was reversed with UV light (360 nm) (Fig. S7a†). Photoswitching of LOGO5 in both directions was stable in the dark over tens of seconds while in current-clamp mode (Fig. 5a) indicating that constant illumination of the sample is not required. This bistability is characteristic of ‘normal’ azobenzenes.^25^ In voltage-clamp mode at −60 mV, blue light (450 nm) illumination induced an outward current (50.3 ± 4.8 pA, *n* = 4 cells), consistent with activation of a potassium conductance (Fig. S7b†). Most importantly when at depolarised potentials, LOGO5 was able to reversibly silence action potential firing under blue light (450 nm) illumination (Fig. 5b). Illuminating with UV light (360 nm) restored action potential firing. The photoswitching of LOGO5 in dissociated rat hippocampal neurons was also highly reliable; photoswitching could be repeated for the entire length that a patch was maintained (∼5–10 minutes; Fig. S7c†), indicating that LOGO5 is a useful tool for experiments over extended periods of time.

**Fig. 6:**
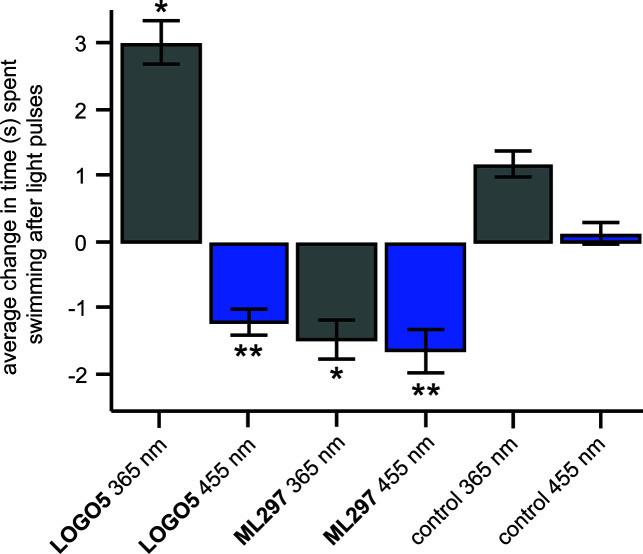
Optical control of zebrafish motility in the presence of LOGO5. After the application of LOGO5 (50 μm), the swimming behaviour of the zebrafish larvae was reduced under blue light illumination and increased under UV light illumination when compared to the control (1% DMSO) zebrafish. Experiments using the non-photoswitchable GIRK opener ML297 (50 μm) showed reduction of the zebrafish motility independent of UV and blue light illumination. (LOGO5*n* = 36 zebrafish; ML297*n* = 36 zebrafish; control (1% DMSO) *n* = 72 zebrafish) (*P < 0.01 *versus* control 365 nm; **P < 0.001 *versus* control 455 nm). Values represent mean ± SEM.

With the control of native GIRK channels accomplished in rat hippocampal neurons, we investigated if LOGO5 had any effect in living animals. We selected zebrafish larvae (*Danio rerio*) as our organism of choice as they are transparent, enabling facile light delivery and they have previously been used in conjunction with biologically active compounds containing azobenzene photoswitches.^26^ Accordingly, zebrafish larvae 5–7 days post fertilisation were exposed to 10 second pulses of UV light (365 nm) and blue light (455 nm), in between interludes of ambient light (Fig. S8†). After the first cycle of UV and blue light pulses the time that the zebrafish larvae spent swimming in the 10 seconds after the light pulse was measured to give the background swimming behaviour. The zebrafish larvae were then incubated with LOGO5 (50 μm) for 1 hour and the same protocol was used to determine the effect of the photochromic GIRK agonist by calculating the change in swimming time. Gratifyingly, the zebrafish larvae showed significantly different changes in swimming time in the presence of LOGO5, which could be modulated by alternating illumination with UV and blue light (Fig. 6). When illuminated with blue light, the zebrafish larvae exhibited reduced swimming times compared to the control experiments. After illuminating with UV light for 10 seconds, the zebrafish larvae dramatically increased the time they spent swimming. However, the zebrafish larvae also showed increased swimming time in the control experiment using only UV light. To dissect the effect of LOGO5 from the native response to UV light, we performed additional experiments using the non-photoswitchable GIRK activator ML297 (Fig. 6). These experiments demonstrated that there is almost no change in the swimming behaviour of the zebrafish larvae when illumination is switched between UV and blue light. This is in stark contrast to the results obtained using LOGO5 and UV light.

## Conclusion

In summary, we have developed a photochromic agonist that enables the optical control of GIRK channels. Our photoswitch, LOGO5, is active as its *trans*-isomer, with UV light illumination converting it to its significantly less active *cis*-isomer. LOGO5 has been shown to work with GIRK channels that bear the GIRK1 subunit and can be used to efficiently reduce neuronal excitability in dissociated hippocampal neurons in a light-dependent manner. LOGO5 can also be implemented *in vivo*, for instance in zebrafish larvae. Future work is focused on the advanced development of LOGO5 as an optical tool to control GIRK channels in higher animals.

## Supplementary Material

SC-007-C5SC04084A-s001
