# Supplementary material for: Optical control of neuronal activity using a light-operated GIRK channel opener (LOGO)
Source: Chem Sci. 2015 Dec 23;7(3):2347–52. doi: 10.1039/c5sc04084a (PMC5234268; doi:10.1039/c5sc04084a)

Electronic Supplementary Material (ESI) for Chemical Science.
This journal is © The Royal Society of Chemistry 2016

Electronic Supplementary Information

Optical control of neuronal activity using a light-operated GIRK channel opener (LOGO)

David M. Barber,[a] Matthias Schönberger,[a] Jessica Burgstaller,[b] Joshua Levitz,[c] C. David Weaver,[d] Ehud Y. Isacoff,[c,e] Herwig Baier[b] and Dirk Trauner*[a]

[a] *Department of Chemistry and Center for Integrated Protein Science, Ludwig Maximilians University Munich, Butenandtstraße 5-13, 81377 Munich, Germany*

[b] *Max Planck Institute of Neurobiology, Am Klopferspitz 18, 82152 Martinsried, Germany*

[c] *Department of Molecular and Cell Biology and Helen Wills Neuroscience Institute, University of California, Berkeley, California, USA*

[d] *Department of Pharmacology and Institute of Chemical Biology, Vanderbilt University School of Medicine, Nashville, Tennessee 37232, USA*

[e] *Physical Bioscience Division, Lawrence Berkeley National Laboratory, Berkeley, California, USA*

dirk.trauner@lmu.de

Contents

**Fig. S1**	**3**
**Fig. S2**	**4**
**Fig. S3 and Fig. S4**	**5**
**Fig. S5 and Fi.g S6**	**6**
**Table S1 and Fig. S7**	**7**
**Fig. S8**	**8**
**Methods**	**9**
**Methods for Chemical Synthesis**	**12**
**Synthesis of LOGO1-6**	**14**
**^{1}H NMR and ^{13}C NMR Spectra**	**29**

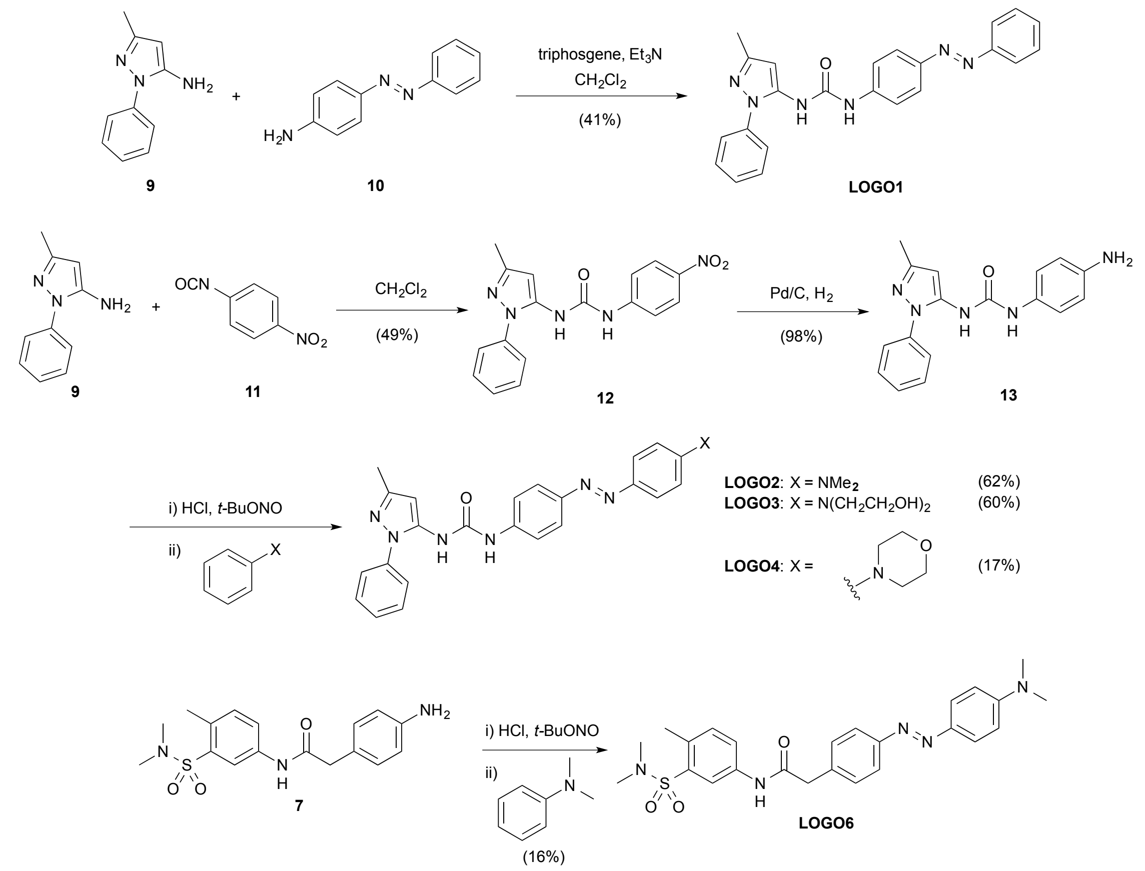

Fig. S1 Synthesis of LOGO1-4 and LOGO6. LOGO1 was prepared by trapping the isocyanate derived from 4-aminoazobenzene (**10**), with the commercially available amino pyrazole **9**. **LOGO2-4** were prepared via azo coupling reactions using aniline **13**. Amino pyrazole **9** was reacted with 4-nitrophenyl isoyanate (**11**) and the resulting nitro urea **12** was subsequently reduced in the presence of Pd/C and hydrogen to furnish aniline **13**. Diazotization of **13** followed by trapping with either *N*,*N*-dimethylaniline, *N*-phenyldiethanolamine or 4-phenylmorpholine afforded **LOGO2**, **LOGO3** and **LOGO4** respectively. **LOGO6** was furnished after an azo coupling reaction between aniline **7** and *N*,*N*-dimethylaniline.

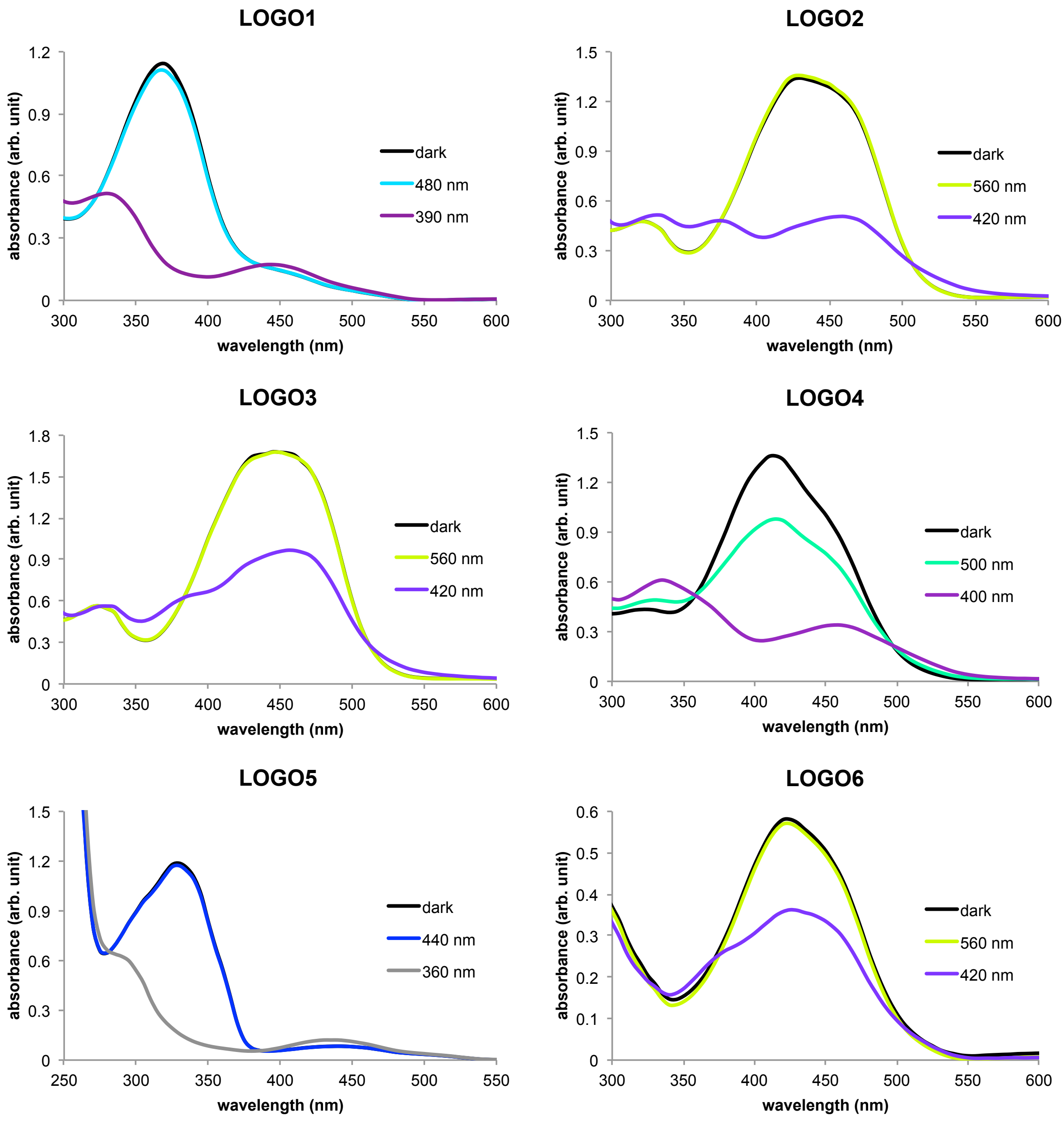

Fig. S2 UV-Vis absorption spectra of LOGO1-6. Solutions of **LOGO1-6** in DMSO (50 μM) were placed in a 1 mL quartz cuvette (10 mm diameter). A light-fibre cable connected to a Till Photonics Polychrome 5000 monochromator was placed in the cuvette until it penetrated the surface of the **LOGO** solution. Illumination was screened from wavelengths 500–340 nm in 10 nm steps (**LOGO1** and **LOGO5**) and 600-340 nm in 20 nm steps (**LOGO2-4** and **LOGO6**) going from higher to lower wavelengths. Every wavelength was applied for 5 min before a UV-Vis spectrum was recorded. Illumination conditions that afforded the highest *trans*-isomer and *cis*-isomer enrichment are shown in Fig. S2.

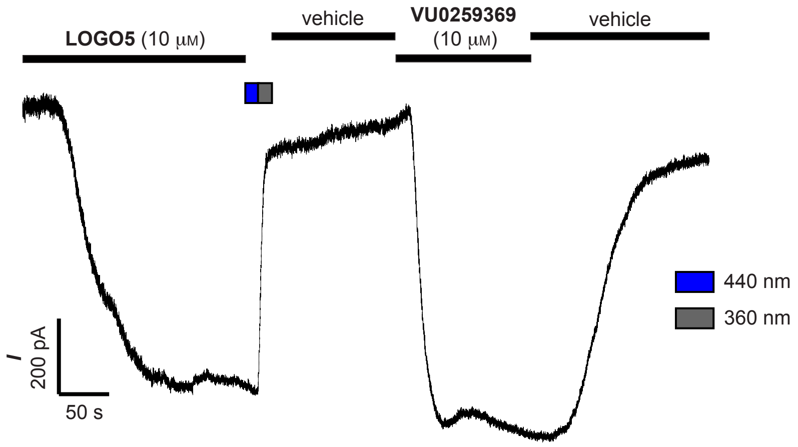

Fig. S3 Comparison of LOGO5 and VU0259369 at GIRK1/2 channels. LOGO5 (10 μM) almost activates GIRK1/2 channels as much as **VU0259369** (10 μM) in the dark and under blue light (440 nm) illumination. Photoswitching using UV light (360 nm) then significantly reduces the activation of the GIRK1/2 channel. Trace representative of $n = 4$ cells.

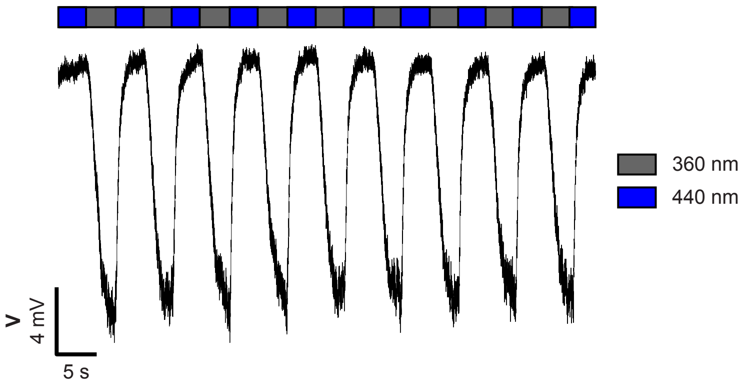

Fig. S4 Current-clamp photoswitching of LOGO5. The photoswitching of **LOGO5** (1 μM) is highly reproducible in current-clamp mode.

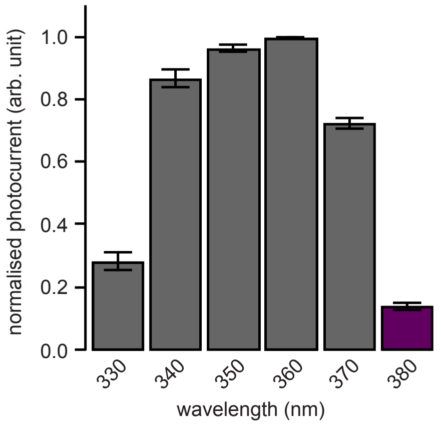

Fig. S5 Action spectrum of LOGO5. It was consistently shown that illuminating **LOGO5** (1 µM) with UV light (360 nm) provided the largest change in current when using blue light (440 nm) as the maximum *trans* wavelength (*n* = 8 cells). Values represent mean ± SEM.

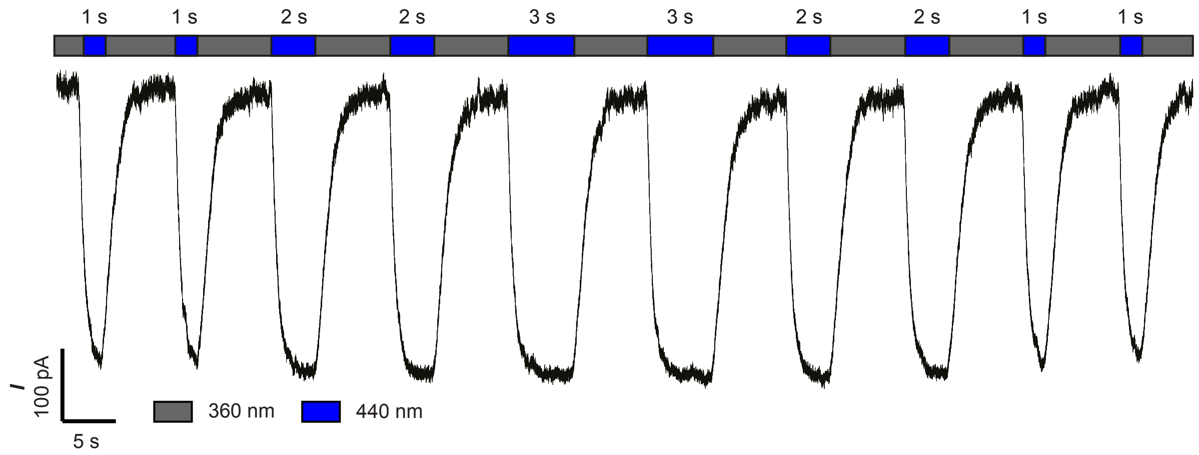

Fig. S6 Time control of GIRK1/2 channels using LOGO5. LOGO5 (5 µM) almost reaches its maximum efficacy after illuminating with blue light (440 nm) for 1 second. Illuminating for 2 seconds enabled **LOGO5** to completely reach its maximum efficacy.

	GIRK1/2				GIRK1/4				GIRK2			
	EC_{50}	SEM	$\%E_{max}$	SEM	EC_{50}	SEM	$\%E_{max}$	SEM	EC_{50}	SEM	$\%E_{max}$	SEM
**LOGO1**	0.7	0.05	71.8	4.2	2.7	0.06	58.4	3.5	–	–	–	–
**LOGO2**	0.6	0.08	37.7	6.0	1.2	0.40	12	2.8	–	–	–	–
**LOGO3**	–	–	–	–	–	–	–	–	–	–	–	–
**LOGO4**	>2.4	ND	>70	ND	>3.9	ND	>35	ND	–	–	–	–
**LOGO5**	1.2	0.09	95	5.0	1.9	0.10	101.5	6.4	–	–	–	–
**LOGO6**	>1.2	ND	>35	ND	>1.3	ND	>16	ND	–	–	–	–

Table S1 Potency, efficacy, and selectivity of LOGO1-6. Shown are the potency and efficacy values obtained from testing **LOGO1-6** using the thallium flux assay on cell lines stably expressing GIRK1/2, GIRK1/4 and GIRK2. Efficacy values were normalised to the maximum activity observed using **ML297** (10 μM) on GIRK1/2 expressing cells. Error values shown are the SEM obtained from three independent experiments. In instances where values are described as greater than a certain value (e.g. **LOGO4** EC_{50} > 2.4 μM), the compound displayed clear concentration-dependent efficacy but either the potency of the compound or the solubility of the compound was too low to obtain an accurate estimate of the potency and efficacy.

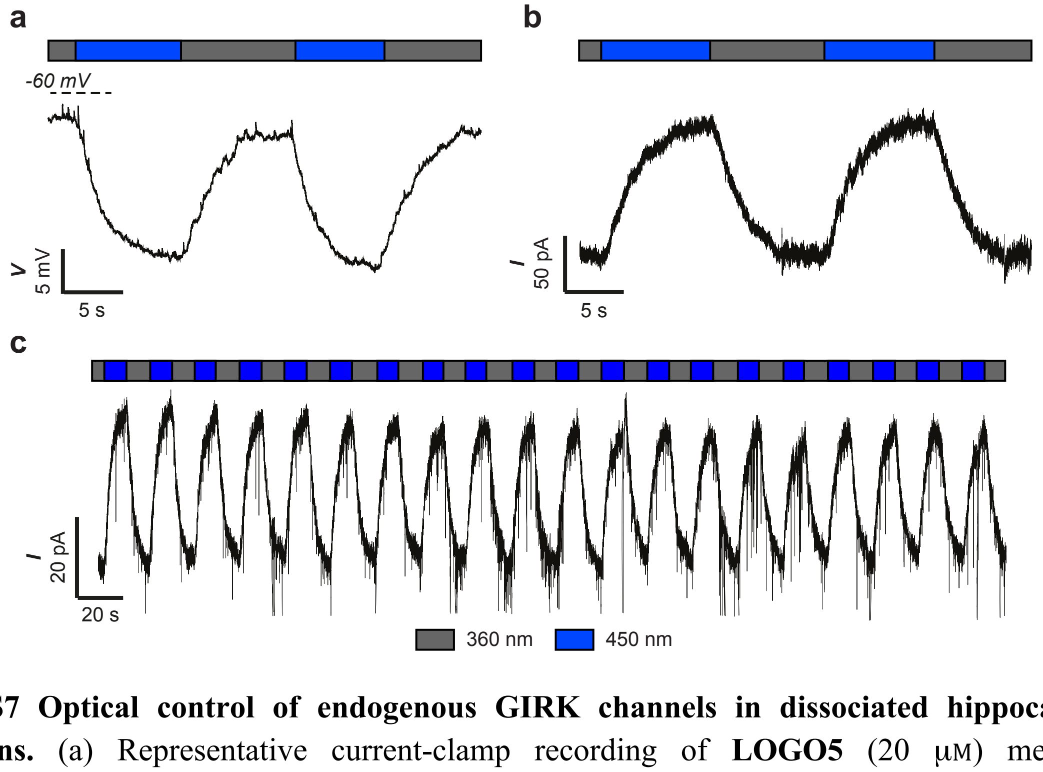

Fig. S7 Optical control of endogenous GIRK channels in dissociated hippocampal neurons. (a) Representative current-clamp recording of **LOGO5** (20 μM) mediated hyperpolarization (15.8 ± 2.5 mV, *n* = 7 cells) in response to blue and UV light. (b) Representative voltage-clamp recording showing outward currents (50.3 ± 4.8 pA, *n* = 4 cells) in response to **LOGO5** photoswitching (holding potential = −60 mV). (c) The photoswitching of **LOGO5** was demonstrated to be highly reversible over extended periods of time.

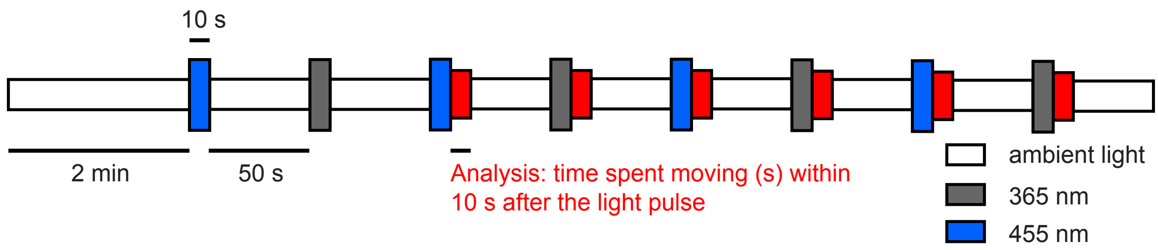

Fig. S8 Protocol used for the evaluation of LOGO5 in zebrafish larvae. The zebrafish larvae were exposed to ambient light for an initial 2 minutes. Then four cycles of blue (455 nm) and UV (365 nm) light pulses were applied for 10 seconds followed by a rest period of 50 seconds. Red indicates the first 10 seconds after the light pulses of the last 3 cycles where the time spent swimming was analysed to calculate the average value for each zebrafish.

Methods

HEK293T Cell Electrophysiology. HEK293T cells were incubated in Dulbecco´s MEM supplemented with 10% FBS and split at 80-90% confluency. For detachment, the growth medium was removed, the cells were washed with calcium free PBS buffer and then treated with trypsin solution 2 min at 37 °C. The detached cells were diluted with growth medium and singularised by pipetting. For transfection, acid-etched coverslips were coated with poly-L-lysin and placed in a 24-well plate. 40,000 cells were added to each well in 500 µL standard growth medium. DNA (per coverslip: 350 ng GIRK1/2 and 50 ng YFP) was mixed with 1 µL polyplus jetprime in 50 µL jetprime buffer. After standing at room temperature for 10-15 min, the DNA-mix was added to the cells shortly after seeding them into the abovementioned 24-well-plate. After 3-5 hours, the medium was exchanged for standard growth medium. Cells were used for electrophysiological recordings 24 hours post transfection.

Whole-cell patch clamp experiments were performed using a standard electrophysiology setup equipped with a HEKA Patch Clamp EPC10 USB amplifier and PatchMaster software (HEKA Electronik). Micropipettes were generated from a Science Products GB200-F-8P with filament pipettes using a vertical puller. Resistance varied between 3-7 MΩ. The extracellular solution contained in mM: 115 NaCl, 50 KCl, 2 $CaCl_2$, 1 $MgCl_2$, 11 glucose and 5 HEPES (KOH to pH 7.4). The intracellular solution contained in mM: 4 NaCl, 107 KCl, 1 $CaCl_2$, 1 $MgCl_2$, 10 EGTA, 2 MgATP, 0.3 Na_2GTP and 5 HEPES (KOH to pH 7.2). The holding potential for voltage-clamp experiments was −60 mV. **LOGO1-6** and the reference agonist **VU0259369** were diluted into the extracellular solution from 100 mM DMSO stock solutions. Illumination during electrophysiology experiments was provided by a TILL Photonics Polychrome 5000 monochromator.

Dissociated Hippocampal Neuron Electrophysiology. Dissociated hippocampal neurons were obtained from postnatal rats (P0-1) and plated at 75,000 cells per coverslip on poly-L-lysine coated glass coverslips (12 mM). Neurons were maintained in medium containing MEM supplemented with 5% FBS, B27 (Invitrogen) and GlutaMAX (Invitrogen).

Whole-cell patch clamp electrophysiology was performed after 10-14 days in vitro using an Axopatch 200B amplifier. The extracellular solution contained in mM: 138 NaCl, 1.5 KCl, 2.5 $CaCl_2$, 1.2 $MgCl_2$, 10 glucose and 5 HEPES (pH 7.4). The intracellular solution contained in mM: 140 potassium gluconate, 10 NaCl, 1 $CaCl_2$, 2 $MgCl_2$, 5 EGTA, 2 MgATP, 0.3 Na_2GTP and 10 HEPES (pH 7.2). Only cells with a resting potential ≤−55 mV were

analysed. The level of 450 nm light induced hyperpolarization in the presence of **LOGO5** (50 μM) was calculated from a baseline of −60 mV. **LOGO5** was diluted into the extracellular solution from a 100 mM DMSO stock solution. pClamp software was used for both data acquisition and control of illumination. Illumination was applied to the entire field of view using a TILL Photonics Polychrome V monochromator through a 20× objective. Power was measured as 0.32 mW at 360 nm and 0.84 mW at 450 nm. The illumination spot size was ~12 mm^2, giving intensity values of ~0.03 mW/mm^2 and ~0.07 mW/mm^2 for 360 and 450 nm, respectively.

Thallium Flux Assays. HEK293 cells co-expressing human GIRK1/2 and GIRK1/4 were constructed as described in Kaufmann *et al.*. For the GIRK2 expressing cell line, HEK293 cells (ATCC) were transfected with GIRK2 in pCMV6-A-puro (Origene) using FuGene 6 (Promega) transfection reagent. All cells were maintained in Minimal Essential Medium, Alpha Medium (Mediatech) supplemented with 10% FBS (Sigma-Aldrich) and GlutaGro (Mediatech), referred to henceforth as cell culture medium. Cell culture medium was further supplemented with selection antibiotics, as appropriate.

Thallium flux assays, test compound solubilisation, serial dilutions and data analysis were performed as described in Kaufmann *et al.* with the following exceptions: the plate reader used was a Panoptic, WaveFront Biosciences, data were acquired at 1 Hz (excitation 480 ± 20 nm, emission 538 ± 20 nm), the thallium stimulus buffer contained in mM: 125 $NaHCO_3$, 1.8 $CaSO_4$, 1 $MgSO_4$, 5 glucose, 2 Tl_2SO_4, 10 HEPES (pH 7.4) and the slopes of vehicle control-subtracted data were calculated from five data points beginning with the point 2 seconds after thallium stimulus buffer addition. The potency and efficacy values depicted in the Fig. 4 and Table S1 were obtained from three independent experiments.

K. Kaufmann, I. Romaine, E. Days, C. Pascual, A. Malik, L. Yang, B. Zou, Y. Du, G. Sliwoski, R. D. Morrison, J. Denton, C. M. Niswender, J. S. Daniels, G. A. Sulikowski, X. Xie, C. W. Lindsley and C. D. Weaver, *ACS Chem. Neurosci.*, 2013, **4**, 1278−1286.

Zebrafish Maintenance and Care. Adult zebrafish (*Danio rerio*) were maintained and bred at 28 °C on a 14/10 hours light/dark cycle. All animal procedures were performed in accordance with the guidelines of the Regierung Oberbayern. All experiments were performed using larvae 5-7 days post fertilisation.

Behavioral Tests in Zebrafish Larva (*Danio rerio*). For experiments, zebrafish larva (*Danio rerio*) at 5-7 days after fertilisation were placed in a 96-well plate at a density of one

animal/well in fish water for video recording. To correct for possible sources of variability, all behavioral assays were performed under carefully controlled experimental conditions including timings, location and setup for stimulation and video recording. 2 minutes of an initial adaptation period were followed by an 8 minute behavioral trial. Light-dependent behavior was induced by changes in illumination between pulses of 10 seconds blue (455 nm) and 10 seconds UV (365 nm) light with interludes of 50 seconds ambient light. Basal motility was measured prior to the addition of **DB125**, **ML297** and DMSO as control. 50 μM of compounds or vehicle (1% DMSO) were added and motility was measured again after 60 minutes incubation. Light cycles were repeated consecutively four times. Zebrafish motility was measured as time spent moving during 10 seconds after the pulses of blue and UV light in the last three cycles of the protocol. Animal tracking was obtained using the Noldus DanioVision system. The action of **DB125**, **ML297** (n = 36 zebrafish) and DMSO (n = 72 zebrafish) was averaged over the declared number of individual fish and quantified as an increase or decrease in time spent moving compared to pre-drug baseline. Illumination was provided by M365L2 (UV 365 nm, 700 mA, 190 mW) and M455L3 (Royal Blue 455 nm, 1000 mA, 900 mW) mounted LEDs driven by a LEDD1B T-Cube LED Driver (1200 mA) using ACL2520U-A lenses (Thorlabs).

^{1}H NMR photoswitching of LOGO5. A sample of **LOGO5** (5 mM in DMSO-d_6) was illuminated in a standard NMR tube using a glass fibre. Monochromatic illumination was switched between 350, 360, 370, 380, 400 and 420 nm, whilst the isomerisation was followed by ^{1}H NMR spectroscopy. Values were recorded after the PSS had been reached.

Methods for Chemical Synthesis

General Experimental Techniques. All reactions were conducted using oven-dried glassware (120 °C) under a positive pressure of nitrogen with magnetic stirring unless otherwise stated. Liquid reagents and solvents were added via syringe or oven-dried stainless steel cannulas through rubber septa. Solids were added under inert gas counter flow or were dissolved in specified solvents prior to addition. Low temperature reactions were carried out in a Dewar vessel filled with the appropriate cooling agent e.g. H_2O/ice (0 °C). Reactions using temperatures above room temperature were conducted using a heated oil bath. Yields refer to spectroscopically pure compounds unless otherwise stated.

Solvents and Reagents. Tetrahydrofuran (THF) was distilled under a nitrogen atmosphere from Na/benzophenone prior to use. Triethylamine (Et_3N) and Hünig's base (DIPEA) were distilled under a nitrogen atmosphere from CaH_2 prior to use. Dry dichloromethane (CH_2Cl_2), *N*,*N*-dimethylformamide (DMF) and methanol (MeOH) were purchased from commercial suppliers and used as received. Solvents for extraction and flash column chromatography were purchased in technical grade purity and distilled under reduced pressure prior to use. All other reagents and solvents were purchased from commercial suppliers and used as received.

Chromatography. Reactions and chromatography fractions were monitored by qualitative thin-layer chromatography (TLC) on silica gel F_{254} TLC plates from Merck KGaA. Analytes on the glass plates were visualized by irradiation with UV light and by immersion of the TLC plate in an appropriate staining solution followed by heating with a hot-air gun. The following staining solutions were applied: Hanessian's (CAM) staining solution [$Ce(SO_4)_2$ (5.0 g), $(NH_4)_6Mo_7O_{24}\cdot 4H_2O$ (25 g), concentrated aqueous H_2SO_4 (50 mL) and H_2O (450 mL)]; potassium permanganate staining solution [$KMnO_4$ (3.0 g), K_2CO_3 (20 g), 5% aqueous NaOH (5.0 mL) and H_2O (300 mL)]; ninhydrin staining solution [ninhydrin (20.0 g) and ethanol (600 mL)]. Flash column chromatography was performed using silica gel, particle size 40 – 63 µm (eluents are given in parenthesis).

Compound Naming. Compound names were generated using ACD/I-Lab software according to standard IUPAC nomenclature.

Melting Points. Melting points were measured on an EZ-Melt apparatus form Stanford Research Systems and are uncorrected.

NMR Spectroscopy. NMR spectra were measured on a Bruker Avance III HD 400 MHz spectrometer equipped with a CryoProbeTM operating at 400 MHz for proton nuclei, 100 MHz for carbon nuclei and 376 MHz for fluorine nuclei. The ^{1}H and ^{13}C NMR shifts are reported in ppm related to the chemical shift of tetramethylsilane. ^{1}H NMR shifts were calibrated to residual solvent resonances: $CDCl_3$ (7.26 ppm), CD_3OD (3.31 ppm), $(CD_3)_2SO$ (2.50 ppm) and $(CD_3)_2CO$ (2.05 ppm). ^{13}C NMR shifts were calibrated to the centre of the multiplet signal of the residual solvent resonance: $CDCl_3$ (77.16 ppm), CD_3OD (49.00 ppm), $(CD_3)_2SO$ (39.52 ppm) and $(CD_3)_2CO$ (29.84 ppm). The ^{19}F NMR shifts are reported in ppm related to the chemical shift of trichlorofluoromethane. ^{1}H NMR spectroscopic data are reported as follows: Chemical shift in ppm (multiplicity, coupling constants, integration). The multiplicities are abbreviated with s (singlet), br s (broad singlet), d (doublet), t (triplet), q (quartet) and m (multiplet). Except for multiplets, the chemical shift of all signals is reported as the centre of the resonance range. Additionally to ^{1}H and ^{13}C NMR measurements, 2D NMR techniques such as homonuclear correlation spectroscopy (COSY), heteronuclear single quantum coherence (HSQC) and heteronuclear multiple bond coherence (HMBC) were used to assist the compound identification process. Coupling constants J are reported in Hz. All raw fid files were processed and the spectra analysed using the program MestReNova 9.0 from Mestrelab Research S. L.

Infrared Spectroscopy. IR spectra were recorded on a PerkinElmer Spectrum BX II FT-IR instrument equipped with an ATR unit. The measured wave numbers are reported in cm^{-1}. Only maximal absorbance's are reported.

Mass Spectrometry. All high-resolution mass spectra (HRMS) were recorded by the LMU Mass Spectrometry Service. HRMS were recorded on a MAT 90 (ESI) spectrometer from Thermo Finnigan GmbH.

UV-Vis Spectroscopy. UV/Vis spectra were recorded on a Varian Cary 50 Scan UV/Vis spectrometer using Helma SUPRASIL precision cuvettes (10 mm light path).

Synthesis and characterisation of 1-(3-methyl-1-phenyl-1*H*-pyrazol-5-yl)-3-(4-nitrophenyl) urea (12)

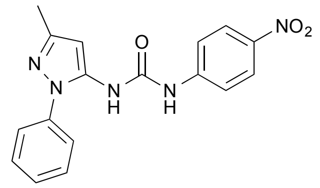

To a solution of compound **9** (1.04 g, 6.00 mmol) in CH_2Cl_2 (50 mL) at r.t. was added compound **11** (1.97 g, 12.0 mmol). The reaction mixture was stirred at r.t. for 1 h and the resulting precipitate was collected by filtration. The collected solid was washed with CH_2Cl_2 (20 mL) and dried under reduced pressure to afford compound **12** (1.98 g, 49%) as a pale yellow solid, which was used in the next step without further purification.

mp: 243 – 246 °C; **TLC** (CH_2Cl_2/MeOH, 9:1): R_f = 0.59 (UV/$KMnO_4$); **^{1}H NMR** (400 MHz, $(CD_3)_2SO$): δ 9.71 (s, 1H), 8.70 (s, 1H), 8.21 – 8.13 (m, 2H), 7.68 – 7.60 (m, 2H), 7.58 – 7.48 (m, 4H), 7.47 – 7.37 (m, 1H), 6.32 (s, 1H), 2.21 (s, 3H); **^{13}C NMR** (100 MHz, $(CD_3)_2SO$): δ 151.3, 148.1, 146.0, 141.3, 138.4, 136.9, 129.4, 127.5, 125.2, 124.3, 117.6, 99.2, 13.8; **IR** (neat): 3264, 1725, 1594, 1543, 1496, 1344, 1328, 1301, 1268, 1186, 1172, 1147, 1108, 1023, 854, 752, 697 cm^{-1}; **HRMS** (ESI, *m/z*): [(M+H)$^+$] calcd. for $C_{17}H_{16}N_5O_3^+$, 338.1248; found 338.1244.

Synthesis and characterisation of 1-(4-aminophenyl)-3-(3-methyl-1-phenyl-1*H*-pyrazol-5-yl)urea (13)

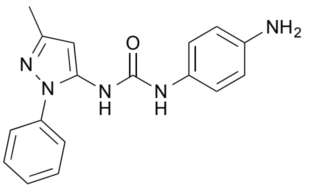

To a solution of compound **12** (1.01 g, 3.00 mmol) in MeOH/EtOAc (1:1, 180 mL) at r.t. was added Pd/C (101 mg, 10 wt. % Pd labelling). The reaction vessel was flushed with hydrogen (×3) and the resulting mixture was stirred at r.t. for 16 h. The reaction mixture was filtered through a pad of Celite® washing with MeOH (50 mL). The filtrate was concentrated under reduced pressure to afford compound **13** (906 mg, 98%) as an off-white solid, which was used in the next step without further purification.

mp: >350 °C; **TLC** (CH_2Cl_2/MeOH, 9:1): R_f = 0.59 (UV/ninhydrin); **^{1}H NMR** (400 MHz, CD_3OD): δ 7.60 – 7.41 (m, 5H), 7.05 (d, *J* = 8.0 Hz, 2H), 6.67 (d, *J* = 8.0 Hz, 2H), 6.31 (s, 1H), 2.27 (s, 3H); **^{13}C NMR** (100 MHz, CD_3OD): δ 154.8, 150.5, 145.3, 139.6, 139.3, 130.7, 130.4, 129.5, 126.5, 123.6 – 123.1 (br m) 117.0, 99.6, 13.7; **IR** (neat): 3436, 3255, 1637, 1595, 1558, 1506, 1461, 1430, 1367, 1302, 1282, 1175, 1023, 804, 772, 689 cm^{-1}; **HRMS** (ESI, *m/z*): [$(M+H)^+$] calcd. for $C_{17}H_{18}N_5O^+$, 308.1506; found 308.1503.

Synthesis and characterisation of 1-(3-methyl-1-phenyl-1*H*-pyrazol-5-yl)-3-{4-[(*E*)-phenyl diazenyl]phenyl}urea (LOGO1)

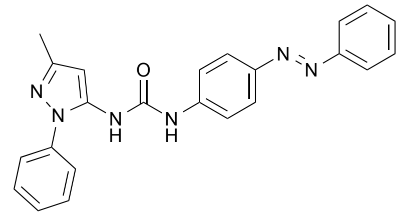

To a solution of compound **10** (197 mg, 1.00 mmol) in CH_2Cl_2 (10 mL) and Et_3N (152 mg, 1.50 mmol, 0.210 mL) at 0 °C was added triphosgene (327 mg, 1.10 mmol). The resulting mixture was allowed to warm to r.t. and then stirred for 24 h. Compound **9** (260 mg, 1.50 mmol) was added and the reaction mixture was stirred at r.t. for 24 h. The reaction mixture was directly purified by flash column chromatography on silica gel eluting with *i*-Hex/EtOAc (6:4) to afford **LOGO1** (163 mg, 41%) as a pale orange solid.

mp: 209 – 211 °C; **TLC** (*i*-Hex/EtOAc, 6:4): R_f = 0.18 (UV/CAM); **^{1}H NMR** (400 MHz, $(CD_3)_2SO$): δ 9.43 (s, 1H), 8.58 (s, 1H), 7.90 – 7.81 (m, 4H), 7.67 – 7.49 (m, 10H), 6.33 (br s, 1H), 2.21 (s, 3H); **^{13}C NMR** (100 MHz, $(CD_3)_2SO$): δ 152.1, 151.3, 148.0, 146.8, 142.7, 138.4, 137.2, 130.9, 129.4, 129.4, 127.4, 124.3, 123.9, 122.3, 118.2, 98.8, 13.8; **IR** (neat): 3272, 1638, 1595, 1557, 1501, 1405, 1296, 1231, 1156, 1024, 855, 769, 754, 687 cm^{-1}; **HRMS** (ESI, *m/z*): $[(M+H)^+]$ calcd. for $C_{23}H_{21}N_6O^+$, 397.1771; found 397.1770; **UV-Vis**: λ_{max} = 370 nm (50 μM in DMSO).

Synthesis and characterisation of 1-(4-{(*E*)-[4-(dimethylamino)phenyl]diazenyl}phenyl)-3-(3-methyl-1-phenyl-1*H*-pyrazol-5-yl)urea (LOGO2)

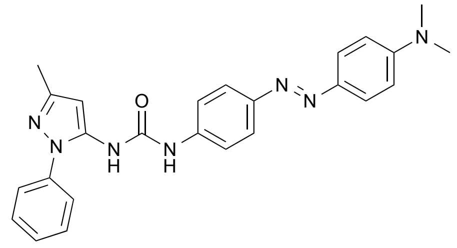

To a suspension of compound **13** (154 mg, 0.500 mmol) in MeOH (5.0 mL) at 0 °C was added conc. HCl (0.25 mL) dropwise. *t*-Butyl nitrite (70 mg, 0.610 mmol, 81 μL, 90% purity) was added dropwise and the resulting solution was stirred at 0 °C for 30 min. The diazonium salt was then added dropwise to a flask containing *N*,*N*-dimethylaniline (67 mg, 0.550 mmol, 69 μL) and NaOAc (246 mg, 3.00 mmol) in MeOH (5.0 mL) at 0 °C. The resulting solution was stirred at 0 °C for 2 h and then at r.t. for 1 h. The reaction was quenched with sat. aq. $NaHCO_3$ (20 mL), diluted with H_2O (20 mL) and extracted with EtOAc (2 × 50 mL). The combined organic extracts were dried over Na_2SO_4, filtered and concentrated under reduced pressure. Purification by flash column chromatography on silica gel eluting with *i*-Hex/EtOAc (1:1) afforded **LOGO2** (137 mg, 62%) as an orange solid.

mp: 230 – 232 °C; **TLC** (*i*-Hex/EtOAc, 1:1): R_f = 0.21 (UV/CAM); **^{1}H NMR** (400 MHz, $(CD_3)_2CO$): δ 8.66 (s, 1H), 7.95 (s, 1H), 7.84 – 7.73 (m, 4H), 7.67 – 7.46 (m, 6H), 7.43 – 7.36 (m, 1H), 6.87 – 6.79 (m, 2H), 6.39 (s, 1H), 3.09 (s, 6H), 2.24 (s, 3H); **^{13}C NMR** (100 MHz, $(CD_3)_2CO$): δ 153.3, 152.1, 149.3, 149.2, 144.28, 142.0, 139.9, 138.1, 130.1, 128.2, 125.3, 125.2, 123.7, 119.3, 112.4, 99.7, 40.3, 14.1; **IR** (neat): 3267, 1637, 1599, 1557, 1501, 1423, 1357, 1297, 1227, 1156, 1141, 943, 819, 755, 692 cm^{-1}; **HRMS** (ESI, *m/z*): [$(M+H)^+$] calcd. for $C_{25}H_{26}N_7O^+$, 440.2193; found 440.2189; **UV-Vis**: λ_{max} = 430 nm (50 μM in DMSO).

Synthesis and characterisation of 1-{4-[(*E*)-{4-[bis(2-hydroxyethyl)amino]phenyl}diazenyl] phenyl}-3-(3-methyl-1-phenyl-1*H*-pyrazol-5-yl)urea (LOGO3)

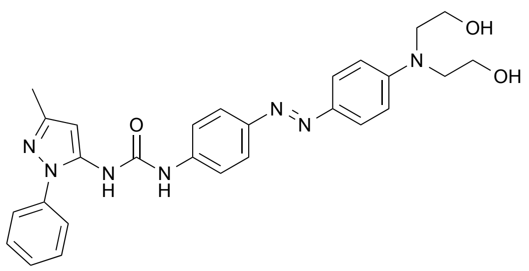

To a suspension of compound **13** (154 mg, 0.500 mmol) in MeOH (5.0 mL) at 0 °C was added conc. HCl (0.25 mL) dropwise. *t*-Butyl nitrite (70 mg, 0.610 mmol, 81 μL, 90% purity) was added dropwise and the resulting solution was stirred at 0 °C for 30 min. The diazonium salt was then added dropwise to a flask containing *N*-phenyldiethanolamine (100 mg, 0.550 mmol) and NaOAc (246 mg, 3.00 mmol) in MeOH (5.0 mL) at 0 °C. The resulting solution was stirred at 0 °C for 2 h and then at r.t. for 2 h. The reaction was quenched with sat. aq. $NaHCO_3$ (20 mL), diluted with H_2O (20 mL) and extracted with EtOAc (2 × 50 mL). The combined organic extracts were dried over Na_2SO_4, filtered and concentrated under reduced pressure. Purification by flash column chromatography on silica gel eluting with CH_2Cl_2/MeOH (19:1 → 9:1) afforded **LOGO3** (150 mg, 60%) as an orange solid.

mp: 205 – 207 °C; **TLC** (CH_2Cl_2/MeOH, 9:1): R_f = 0.53 (UV/CAM); **^{1}H NMR** (400 MHz, CD_3OD): δ 7.80 – 7.70 (m, 4H), 7.60 – 7.43 (m, 7H), 6.88 – 6.81 (m, 2H), 6.39 (s, 1H), 3.77 (t, *J* = 6.0 Hz, 4H), 3.65 (t, *J* = 6.0 Hz, 4H), 2.28 (s, 3H); **^{13}C NMR** (100 MHz, CD_3OD): δ 153.6, 151.9, 150.6, 150.0, 144.8, 141.6, 139.3, 139.3, 130.7, 129.7, 126.6, 125.8, 124.0, 120.1, 112.7, 99.5, 60.3, 55.0, 13.7; **IR** (neat): 3047, 1716, 1597, 1539, 1498, 1386, 1343, 1297, 1259, 1196, 1152, 1074, 1001, 846, 820, 756 cm^{-1}; **HRMS** (ESI, *m/z*): [$(M+H)^+$] calcd. for $C_{27}H_{30}N_7O_3^+$, 500.2405; found 500.2399; **UV-Vis**: λ_{max} = 445 nm (50 μM in DMSO).

Synthesis and characterisation of 1-(3-methyl-1-phenyl-1*H*-pyrazol-5-yl)-3-(4-{(*E*)-[4-(morpholin-4-yl)phenyl]diazenyl}phenyl)urea (LOGO4)

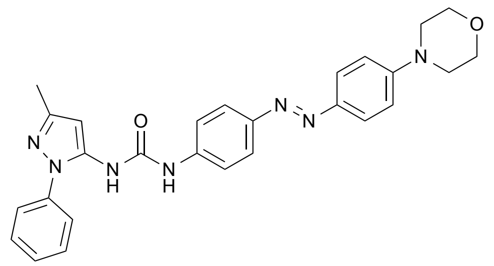

To a suspension of compound **13** (154 mg, 0.500 mmol) in MeOH (5.0 mL) at 0 °C was added conc. HCl (0.25 mL) dropwise. *t*-Butyl nitrite (70 mg, 0.610 mmol, 81 µL, 90% purity) was added dropwise and the resulting solution was stirred at 0 °C for 30 min. The diazonium salt was then added dropwise to a flask containing 4-phenylmorpholine (98 mg, 0.600 mmol) and pyridine (198 mg, 2.50 mmol, 202 µL) in MeOH (5.0 mL) at 0 °C. The resulting solution was stirred at 0 °C for 4 h and then at r.t. for 24 h. The reaction mixture was concentrated under reduced pressure and the residue was purified by flash column chromatography on silica gel eluting with EtOAc/*i*-Hex (6:4) to afford **LOGO4** (41 mg, 17%) as an orange solid.

mp: 245 – 248 °C decomp; **TLC** (EtOAc/*i*-Hex, 6:4): R_f = 0.29 (UV/CAM); **^{1}H NMR** (400 MHz, $(CD_3)_2SO$): δ 9.36 (s, 1H), 8.57 (s, 1H), 7.85 – 7.70 (m, 3H), 7.65 – 7.49 (m, 6H), 7.47 – 7.35 (m, 2H), 7.14 – 6.99 (m, 2H), 6.32 (s, 1H), 3.85 – 3.63 (m, 4H), 3.32 – 3.17 (m, 4H), 2.21 (s, 3H); **^{13}C NMR** (100 MHz, $(CD_3)_2SO$): δ 152.8, 151.4, 148.0, 147.2, 144.5, 141.5, 138.4, 137.3, 129.4, 127.4, 124.3, 124.0, 123.2, 118.2, 114.1, 98.7, 65.9, 47.2, 13.9; **IR** (neat): 3305, 2923, 1723, 1650, 1597, 1545, 1500, 1447, 1379, 1311, 1264, 1228, 1196, 1156, 1113, 1023, 924, 848, 824, 760 cm^{-1}; **HRMS** (ESI, *m/z*): $[(M+H)^+]$ calcd. for $C_{27}H_{28}N_7O_2^+$, 482.2299; found 482.2297; **UV-Vis**: λ_{max} = 415 nm (50 µM in DMSO).

Synthesis and characterisation of *N*,*N*-2-trimethyl-5-nitrobenzenesulfonamide (3)

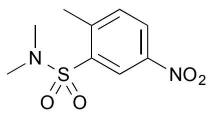

To a solution of compound **2** (3.53 g, 15.0 mmol) in THF (300 mL) at 0 °C was added a solution of dimethylamine (45 mL, 40 wt. % in H_2O). The resulting mixture was stirred at 0 °C for 2 h and then concentrated under reduced pressure. The resulting residue was diluted with H_2O (200 mL) and extracted with EtOAc (2 × 200 mL). The combined organic extracts were dried over Na_2SO_4, filtered and concentrated under reduced pressure to afford compound **3** as a pale yellow oil, which was used in the next step without further purification.

TLC (*i*-Hex/EtOAc, 1:1): R_f = 0.58 (UV/$KMnO_4$); **^{1}H NMR** (400 MHz, $CDCl_3$): δ 8.69 (d, *J* = 2.5 Hz, 1H), 8.29 (dd, *J* = 8.5, 2.5 Hz, 1H), 7.52 (d, *J* = 8.5 Hz, 1H), 2.89 (s, 6H), 2.74 (s, 3H); **^{13}C NMR** (100 MHz, $CDCl_3$): δ 146.1, 145.7, 138.5, 134.1, 127.0, 124.8, 37.4, 21.1; **IR** (neat): 3109, 2938, 1604, 1585, 1522, 1474, 1459, 1346, 1326, 1265, 1157, 1123, 1057, 954, 891, 730 cm^{-1}; **HRMS** (ESI, *m/z*): [(M−H)$^-$] calcd. for $C_9H_{11}N_2O_4S^-$, 243.0445; found 243.0444.

Synthesis and characterisation of 5-amino-*N,N*-2-trimethylbenzenesulfonamide (4)

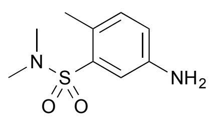

To a solution of compound **3** in a mixture of MeOH/EtOAc (1:1, 200 mL) at r.t. under an atmosphere of nitrogen was added Pd/C (366 mg, 10 wt. % Pd labelling). The reaction vessel was then flushed with hydrogen (×3) and the resulting mixture was stirred at r.t. for 16 h. The reaction mixture was filtered through a plug of Celite® washing with MeOH (100 mL). The filtrate was concentrated under reduced pressure to afford compound **4** (3.17 g, 99% over two steps) as a pale yellow oil. On standing the product crystallised into a pale yellow solid, which was used in the next step without further purification.

mp: 81 – 83 °C; **TLC** (*i*-Hex/EtOAc, 1:1): R_f = 0.47 (UV/ninhydrin); ^{1}**H NMR** (400 MHz, CD_3OD): δ 7.19 (d, *J* = 2.5 Hz, 1H), 7.07 (d, *J* = 8.0 Hz, 1H), 6.82 (dd, *J* = 8.0, 2.5 Hz, 1H), 2.73 (s, 6H), 2.42 (s, 3H); ^{13}**C NMR** (100 MHz, CD_3OD): δ 147.5, 136.5, 134.6, 126.7, 120.4, 116.8, 37.5, 19.9; **IR** (neat): 3452, 3364, 2975, 1632, 1613, 1501, 1451, 1318, 1301, 1160, 1138, 1058, 1047, 1036, 947, 821, 726, 697, 682 cm^{-1}; **HRMS** (ESI, *m/z*): [$(M+H)^+$] calcd. for $C_9H_{15}N_2O_2S^+$, 215.0849; found 215.0847.

Synthesis and characterisation of *N*-[3-(dimethylsulfamoyl)-4-methylphenyl]-2-(4-nitro phenyl)acetamide (6)

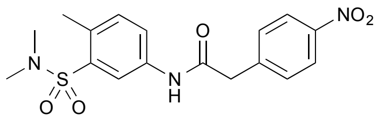

To a suspension of compound **5** (375 mg, 2.07 mmol) in CH_2Cl_2 (10 mL) at 0 °C was added a solution of oxalyl chloride (1.08 mL, 2.0 M in CH_2Cl_2) and DMF (4 drops). The resulting mixture was warmed to r.t., stirred for 30 min and then concentrated under a stream of nitrogen. The residue was dissolved in DMF (10 mL) and then added dropwise to a solution of compound **4** (386 mg, 1.80 mmol) in DMF (10 mL) and DIPEA (465 mg, 3.60 mmol, 627 μL) at 0 °C. The resulting mixture was warmed to r.t. and stirred for 20 h. The reaction mixture was diluted with H_2O (100 mL) and extracted with EtOAc (100 mL). The organic extract was washed with a mixture of H_2O/brine (9:1, 4 × 100 mL), dried over Na_2SO_4, filtered and concentrated under reduced pressure. Purification by flash column chromatography on silica gel eluting with *i*-Hex/EtOAc (1:1) afforded compound **6** (380 mg, 56%) as a yellow oil. On standing the product crystallised into an off-white solid.

mp: 180 – 182 °C; **TLC** (*i*-Hex/EtOAc, 1:1): R_f = 0.28 (UV/$KMnO_4$); **^{1}H NMR** (400 MHz, CD_3OD): δ 8.27 – 8.18 (m, 2H), 8.15 (d, *J* = 2.5 Hz, 1H), 7.69 (dd, *J* = 8.5, 2.5 Hz, 1H), 7.64 – 7.56 (m, 2H), 7.34 (d, *J* = 8.5 Hz, 1H), 3.85 (s, 2H), 2.80 (s, 6H), 2.55 (s, 3H); **^{13}C NMR** (100 MHz, CD_3OD): δ 170.9, 148.5, 144.2, 138.2, 137.4, 134.5, 134.4, 131.5, 124.8, 124.6, 121.8, 44.0, 37.5, 20.3; **IR** (neat): 3378, 2930, 1681, 1607, 1520, 1494, 1434, 1386, 1346, 1159, 1142, 957, 731 cm^{-1}; **HRMS** (ESI, *m/z*): $[(M+H)^+]$ calcd. for $C_{17}H_{20}N_3O_5S^+$, 378.1118; found 337.1115.

Synthesis and characterisation of 2-(4-aminophenyl)-*N*-[3-(dimethylsulfamoyl)-4-methyl phenyl]acetamide (7)

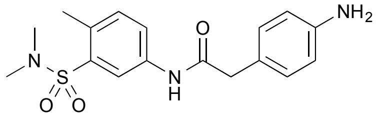

To a solution of compound **6** (340 mg, 0.900 mmol) in a mixture of MeOH/EtOAc (2:1, 27 mL) at r.t. was added Pd/C (34 mg, 10 wt. % Pd labelling). The reaction vessel was flushed with hydrogen (×3) and the resulting mixture was stirred at r.t. for 16 h. The reaction mixture was filtered through a plug of Celite® washing with MeOH (30 mL) and the filtrate was concentrated under reduced pressure to afford compound **7** (261 mg, 83%) as a pale yellow oil, which was used in the next step without further purification.

TLC (EtOAc/*i*-Hex, 3:1): $R_f = 0.32$ (UV/ninhydrin); **^{1}H NMR** (400 MHz, CD_3OD): δ 8.13 (d, $J = 2.5$ Hz, 1H), 7.66 (dd, $J = 8.5, 2.5$ Hz, 1H), 7.29 (d, $J = 8.5$ Hz, 1H), 7.12 – 7.05 (m, 2H), 6.72 – 6.65 (m, 2H), 3.52 (s, 2H), 2.77 (s, 6H), 2.53 (s, 3H); **^{13}C NMR** (100 MHz, CD_3OD): δ 173.3, 147.6, 138.3, 137.3, 134.4, 134.1, 130.8, 125.8, 124.9, 121.7, 116.8, 44.0, 37.6, 20.3; **IR** (thin film): 3354, 2930, 1667, 1625, 1608, 1590, 1515, 1490, 1456, 1388, 1307, 1255, 1139, 1059, 956, 831, 732 cm^{-1}; **HRMS** (ESI, *m/z*): [$(M+H)^+$] calcd. for $C_{17}H_{22}N_3O_3S^+$, 348.1376; found 348.1375.

Synthesis and characterisation of *N*-[3-(dimethylsulfamoyl)-4-methylphenyl]-2-{4-[(*E*)-phenyldiazenyl]phenyl}acetamide (*trans*-LOGO5)

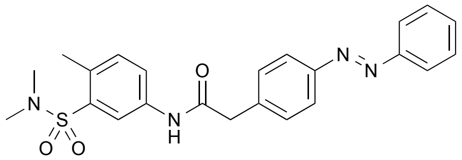

To a solution of compound **7** (87 mg, 0.250 mmol) in CH_2Cl_2/AcOH (1:1, 6 mL) at r.t. was added compound **8** (134 mg, 1.25 mmol). The resulting mixture was stirred at r.t. for 16 h and then concentrated under reduced pressure. Purification by flash column chromatography on silica gel eluting with *i*-Hex/EtOAc (1:1) afforded ***trans*-LOGO5** (95 mg, 87%) as an orange oil.

TLC (*i*-Hex/EtOAc, 1:1): R_f = 0.29 (UV/$KMnO_4$); **^{1}H NMR** (400 MHz, $(CD_3)_2SO$): δ 10.54 (s, 1H), 8.11 (s, 1H), 7.93 – 7.84 (m, 4H), 7.82 – 7.74 (m, 1H), 7.64 – 7.52 (m, 5H), 7.38 (d, *J* = 8.5 Hz, 1H), 3.79 (s, 2H), 2.72 (s, 6H), 2.48 (s, 3H); **^{13}C NMR** (100 MHz, $(CD_3)_2SO$): δ 169.0, 151.9, 150.8, 139.5, 137.4, 135.5, 133.4, 131.5, 131.4, 130.4, 129.5, 122.9, 122.6, 122.5, 119.5, 43.0, 36.9, 19.7; **IR** (neat): 3314, 2930, 1666, 1591, 1526, 1490, 1388, 1322, 1255, 1156, 1141, 1059, 956, 829, 768, 730, 688 cm^{-1}; **HRMS** (ESI, *m/z*): $[(M+H)^+]$ calcd. for $C_{23}H_{25}N_4O_3S^+$, 437.1642; found 437.1639; **UV-Vis**: λ_{max} = 330 nm (50 μM in DMSO).

Synthesis and characterisation of *N*-[3-(dimethylsulfamoyl)-4-methylphenyl]-2-{4-[(*Z*)-phenyldiazenyl]phenyl}acetamide (*cis*-LOGO5)

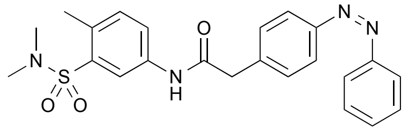

A sample of ***cis*-LOGO5** for NMR characterisation was prepared by illuminating a solution of ***trans*-LOGO5** (5 mM in $(CD_3)_2SO$) with UV light (360 nm) while it was in the NMR spectrometer. The UV light (360 nm) was delivered to the NMR tube via a fibre optic cable that was attached to a monochromator. The sample was illuminated for 14 h.

^{1}H NMR (400 MHz, $(CD_3)_2SO$): δ 10.40 (s, 1H), 8.05 (s, 1H), 7.77 – 7.68 (m, 1H), 7.41 – 7.08 (m, 6H), 6.90 – 6.72 (m, 4H), 3.60 (s, 2H), 2.71 (s, 6H), 2.47 (m, 3H); **^{13}C NMR** (100 MHz, $(CD_3)_2SO$): δ 169.0, 153.7, 152.0, 137.3, 135.5, 134.7, 133.3, 131.4, 129.6, 128.9, 127.1, 122.8, 120.1, 119.7, 119.4, 42.5, 36.9, 19.6.

Synthesis and characterisation of 2-(4-{(*E*)-[4-(dimethylamino)phenyl]diazenyl}phenyl)-*N*-[3-(dimethylsulfamoyl)-4-methylphenyl]acetamide (LOGO6)

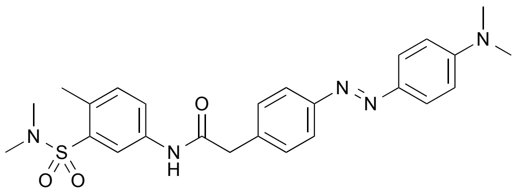

To a solution of compound **7** (70 mg, 0.200 mmol) in MeOH (2.0 mL) at 0 °C was added conc. HCl (0.05 mL) dropwise. *t*-Butyl nitrite (25 mg, 0.220 mmol, 29 µL, 90% purity) was added dropwise and the resulting solution was stirred at 0 °C for 30 min. The diazonium salt was then added dropwise to a flask containing a solution of *N*,*N*-dimethylaniline (27 mg, 0.220 mmol, 28 µL) and NaOAc (98 mg, 1.20 mmol) in MeOH (2.0 mL) at 0 °C. The resulting solution was stirred at 0 °C for 4 h and then at r.t. for 16 h. The reaction was quenched with sat. aq. $NaHCO_3$ (10 mL), diluted with H_2O (10 mL) and extracted with EtOAc (2 × 40 mL). The combined organic extracts were dried over Na_2SO_4, filtered and concentrated under reduced pressure. Purification by flash column chromatography on silica gel eluting with *i*-Hex/EtOAc (1:1) afforded **LOGO6** (15 mg, 16%) as an orange oil.

TLC (*i*-Hex/EtOAc, 1:1): R_f = 0.34 (UV/CAM); **^{1}H NMR** (400 MHz, $CDCl_3$): δ 7.93 – 7.84 (m, 4H), 7.82 (d, *J* = 2.0 Hz, 1H), 7.70 (dd, *J* = 8.5, 2.0 Hz, 1H), 7.44 (d, *J* = 8.0 Hz, 2H), 7.40 (br s, 1H), 7.23 (d, *J* = 8.5 Hz, 1H), 6.76 (d, *J* = 8.5 Hz, 2H), 3.80 (s, 2H), 3.10 (s, 6H), 2.81 (s, 6H), 2.55 (s, 3H); **^{13}C NMR** (100 MHz, $CDCl_3$): δ 169.3, 152.9, 152.7, 143.7, 136.4, 135.9, 135.0, 133.8, 133.5, 130.3, 125.3, 124.1, 123.2, 120.9, 111.6, 44.7, 40.5, 37.4, 20.2; **IR** (thin film): 3326, 2925, 1666, 1600, 1518, 1490, 1445, 1388, 1364, 1312, 1157, 1139, 947, 824, 732 cm^{-1}; **HRMS** (ESI, *m/z*): [$(M+H)^+$] calcd. for $C_{25}H_{30}N_5O_3S^+$, 480.2064; found 480.2062; **UV-Vis**: λ_{max} = 425 nm (50 µM in DMSO).

Synthesis and characterisation of 2-(2-chlorophenyl)-*N*-[3-(dimethylsulfamoyl)-4-methyl phenyl]acetamide (VU0259369)

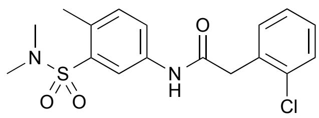

To a suspension of 2-chlorophenylacetic acid (98 mg, 0.575 mmol) in CH_2Cl_2 (2.5 mL) at 0 °C was added a solution of oxalyl chloride (0.300 mL, 0.600 mmol, 2.0 M in CH_2Cl_2) and DMF (2 drops). The resulting mixture was warmed to r.t., stirred for 30 min and then concentrated under a stream of nitrogen. The residue was dissolved in DMF (2.5 mL) and then added dropwise to a solution of compound **4** (107 mg, 0.500 mmol) in DMF (2.5 mL) and DIPEA (129 mg, 1.00 mmol, 174 μL) at 0 °C. The resulting mixture was warmed to r.t. and stirred for 16 h. The reaction mixture was diluted with H_2O (25 mL) and extracted with Et_2O (2 × 25 mL). The combined organic extracts were washed with H_2O (2 × 25 mL), dried over Na_2SO_4, filtered and concentrated under reduced pressure. Purification by flash column chromatography on silica gel eluting with *i*-Hex/EtOAc (6:4) afforded **VU0259369** (124 mg, 68%) as a colourless oil.

TLC (*i*-Hex/EtOAc, 1:1): R_f = 0.50 (UV/$KMnO_4$); **^{1}H NMR** (400 MHz, $CDCl_3$): δ 7.94 (br s, 1H), 7.85 – 7.75 (m, 2H), 7.42 – 7.31 (m, 2H), 7.27 – 7.16 (m, 3H), 3.82 (s, 2H), 2.77 (s, 6H), 2.52 (s, 3H); **^{13}C NMR** (100 MHz, $CDCl_3$): δ 168.5, 136.2, 136.0, 134.5, 133.5, 133.4, 132.6, 131.9, 129.9, 129.3, 127.5, 124.3, 121.1, 42.2, 37.3, 20.2; **IR** (neat): 3443, 2996, 2913, 1664, 1596, 1533, 1490, 1436, 1406, 1310, 1156, 1142, 930, 896, 731, 697, 667 cm^{-1}; **HRMS** (ESI, *m/z*): [$(M+H)^+$] calcd. for $C_{17}H_{20}ClN_2O_3S^+$, 367.0878; found 367.0883.

Synthesis and characterisation of 1-(3,4-difluorophenyl)-3-(3-methyl-1-phenyl-1*H*-pyrazol-5-yl)urea (ML297)

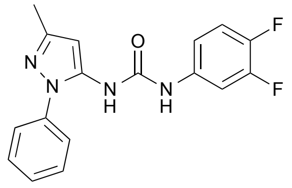

To a solution of compound **2** (1.12 g, 6.45 mmol) in CH_2Cl_2 (26 mL) at r.t. was added 3,4-difluorophenyl isocyanate (1.58 g, 10.2 mmol, 1.19 mL). The resulting mixture was stirred at r.t. for 2 h. The white precipitate was collected by filtration washing with CH_2Cl_2 (50 mL) and dried under reduced pressure to afford **ML297** (1.89 g, 80%) as a white solid, which was used without further purification.

mp: 203 – 204 °C; **^{1}H NMR** (400 MHz, $(CD_3)_2SO$): δ 9.20 (s, 1H), 8.51 (s, 1H), 7.60 (ddd, *J* = 13.5, 7.5, 2.5 Hz, 1H), 7.55 – 7.46 (m, 4H), 7.44 – 7.37 (m, 1H), 7.36 – 7.25 (m, 1H), 7.11 – 7.02 (m, 1H), 6.27 (s, 1H), 2.19 (s, 3H); **^{13}C NMR** (100 MHz, $(CD_3)_2SO$): δ 151.8, 149.2 (dd, *J* = 242.5, 13.0 Hz), 148.1, 144.7 (dd, *J* = 240.0, 12.5 Hz), 138.5, 137.3, 136.6 (dd, *J* = 9.5, 2.5 Hz), 129.5, 127.5, 124.3, 117.6 (d, *J* = 18.0 Hz), 114.6 (dd, *J* = 6.0, 3.0 Hz), 107.3 (d, *J* = 22.0 Hz), 99.3, 13.9; **^{19}F NMR** (376 MHz, $(CD_3)_2SO$): δ –137.3 (d, *J* = 23.0 Hz), –146.6 (d, *J* = 23.0 Hz); **IR** (neat): 3268, 1641, 1621, 1571, 1515, 1502, 1429, 1292, 1269, 1236, 1216, 1185, 1116, 1023, 964, 863, 805, 786, 763, 746 cm^{-1}; **HRMS** (ESI, *m/z*): $[(M+H)^+]$ calcd. for $C_{17}H_{15}F_2N_4O^+$, 329.1208; found 329.1210.

^{1}H NMR spectrum of compound **12**

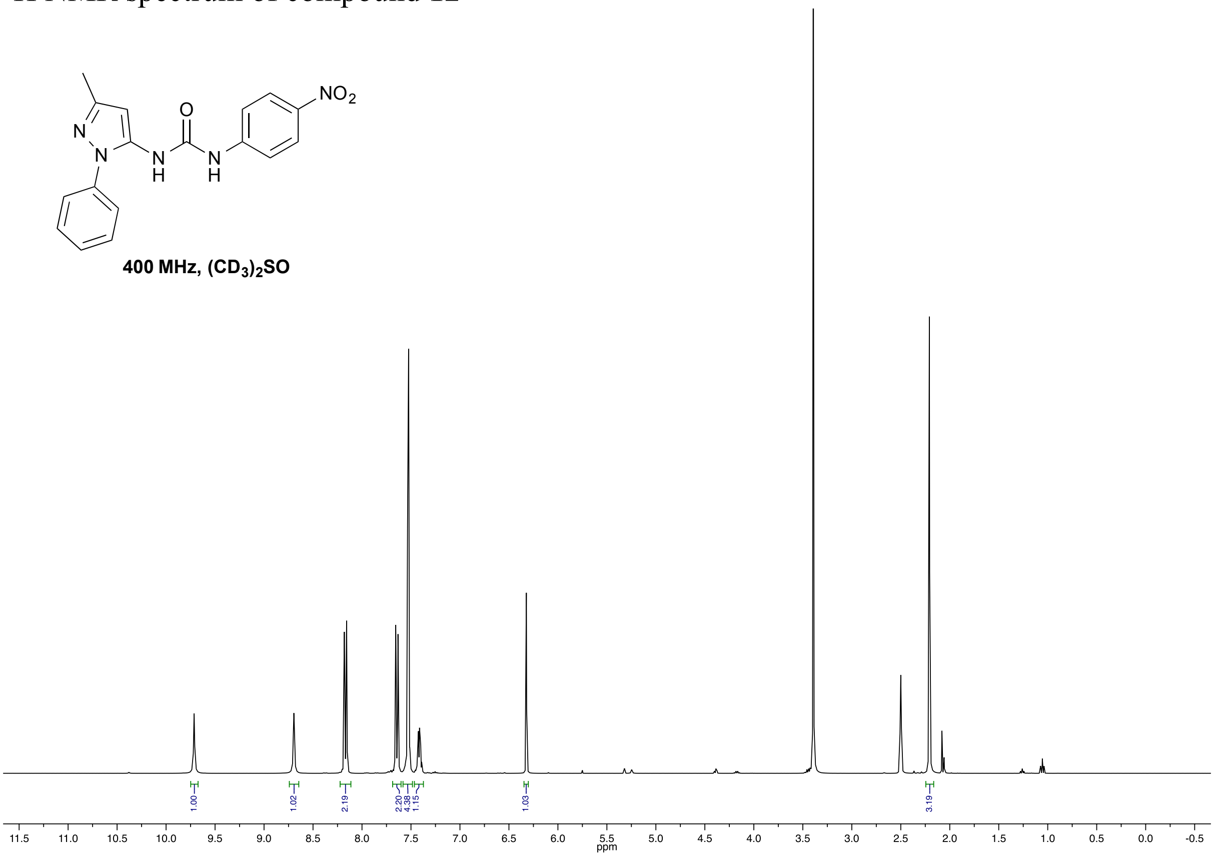

^{13}C NMR spectrum of compound **12**

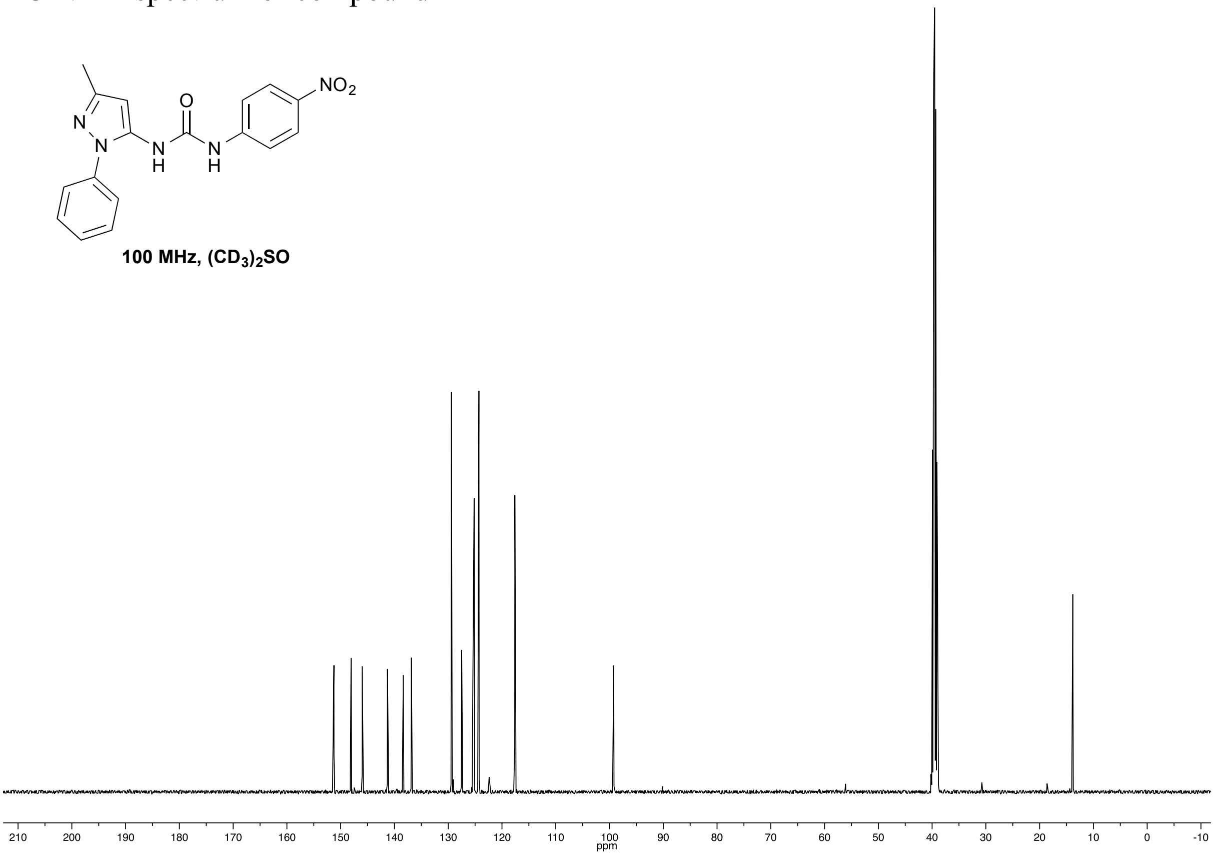

^{1}H NMR spectrum of compound **13**

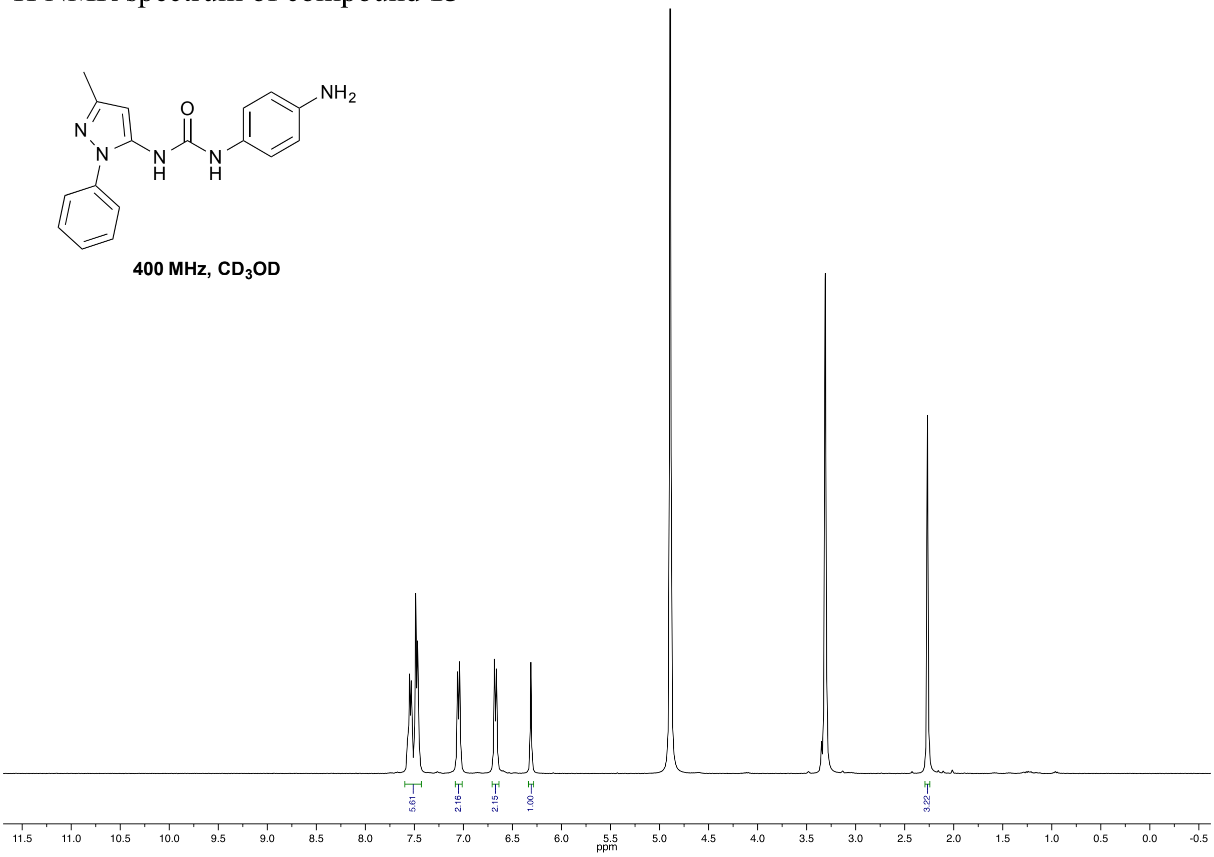

^{13}C NMR spectrum of compound **13**

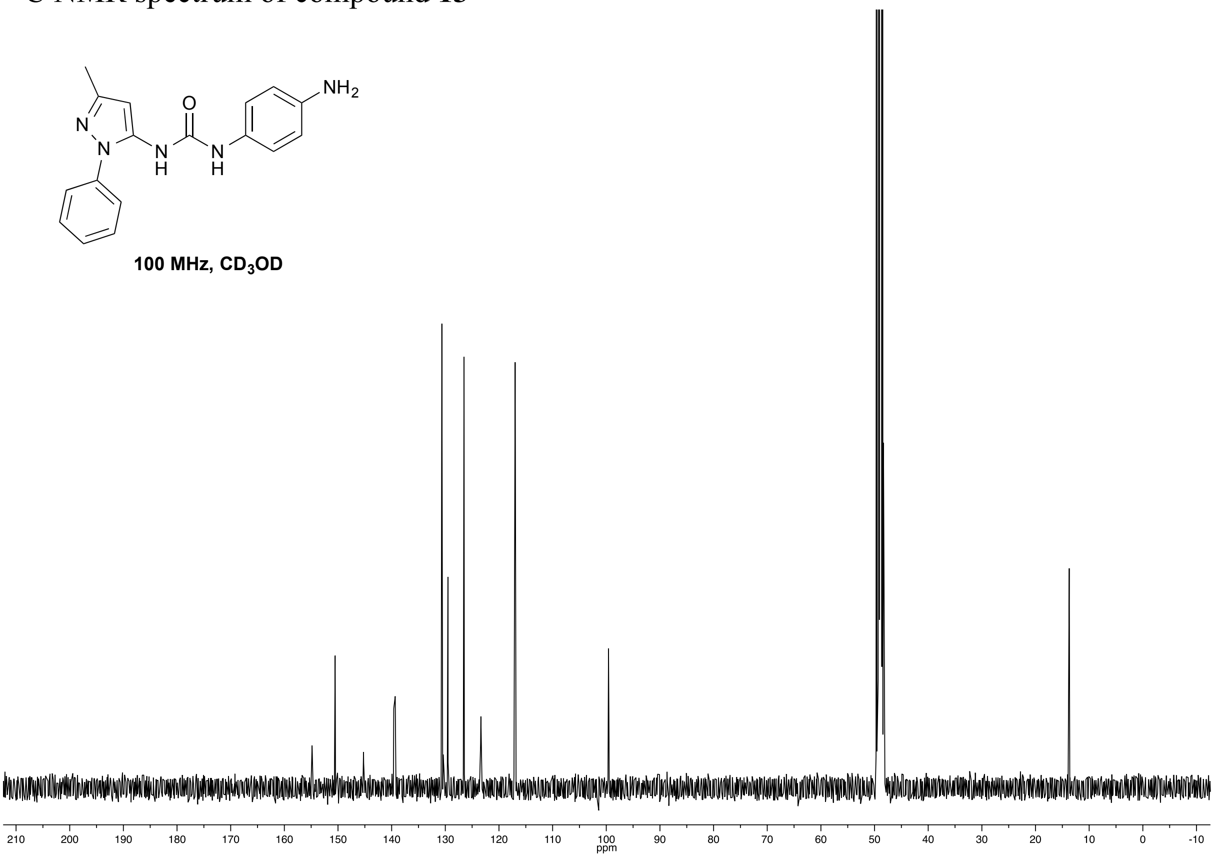

^{1}H NMR spectrum of **LOGO1**

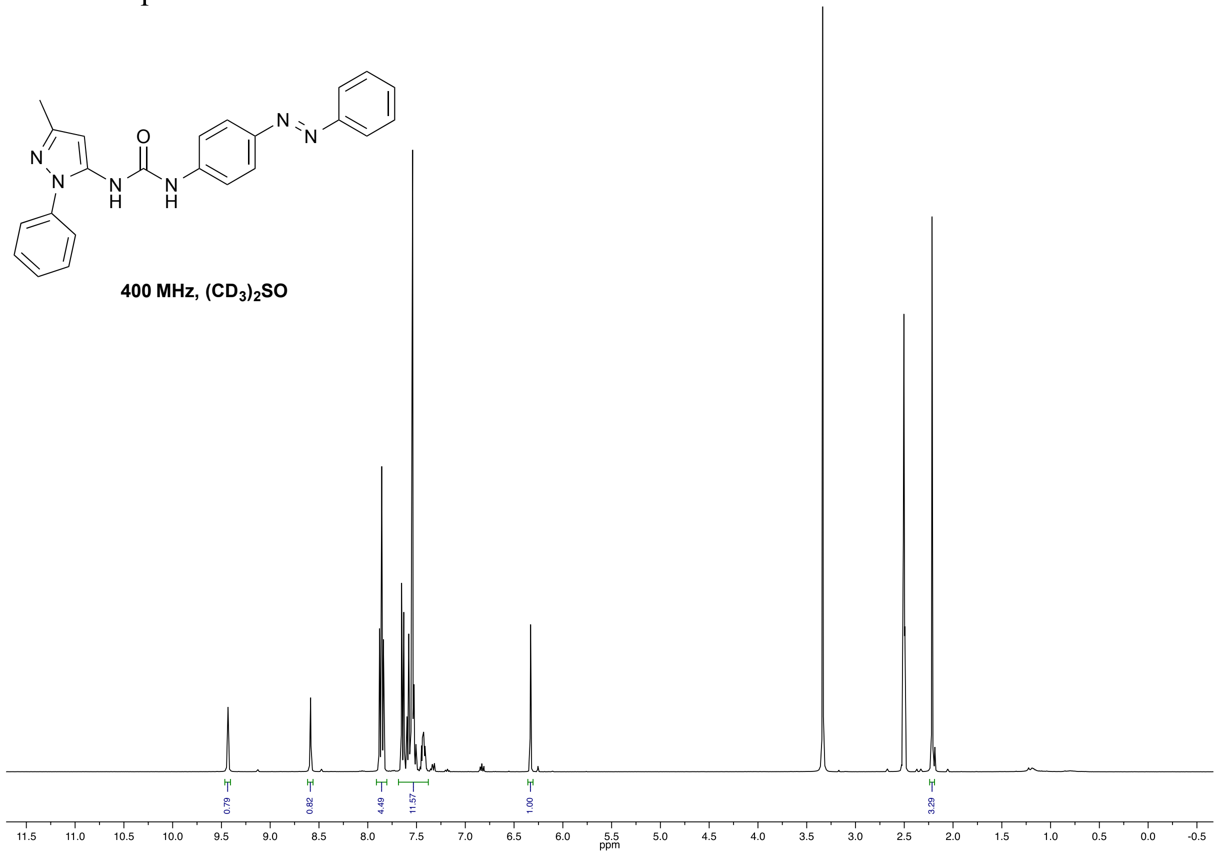

^{13}C NMR spectrum of **LOGO1**

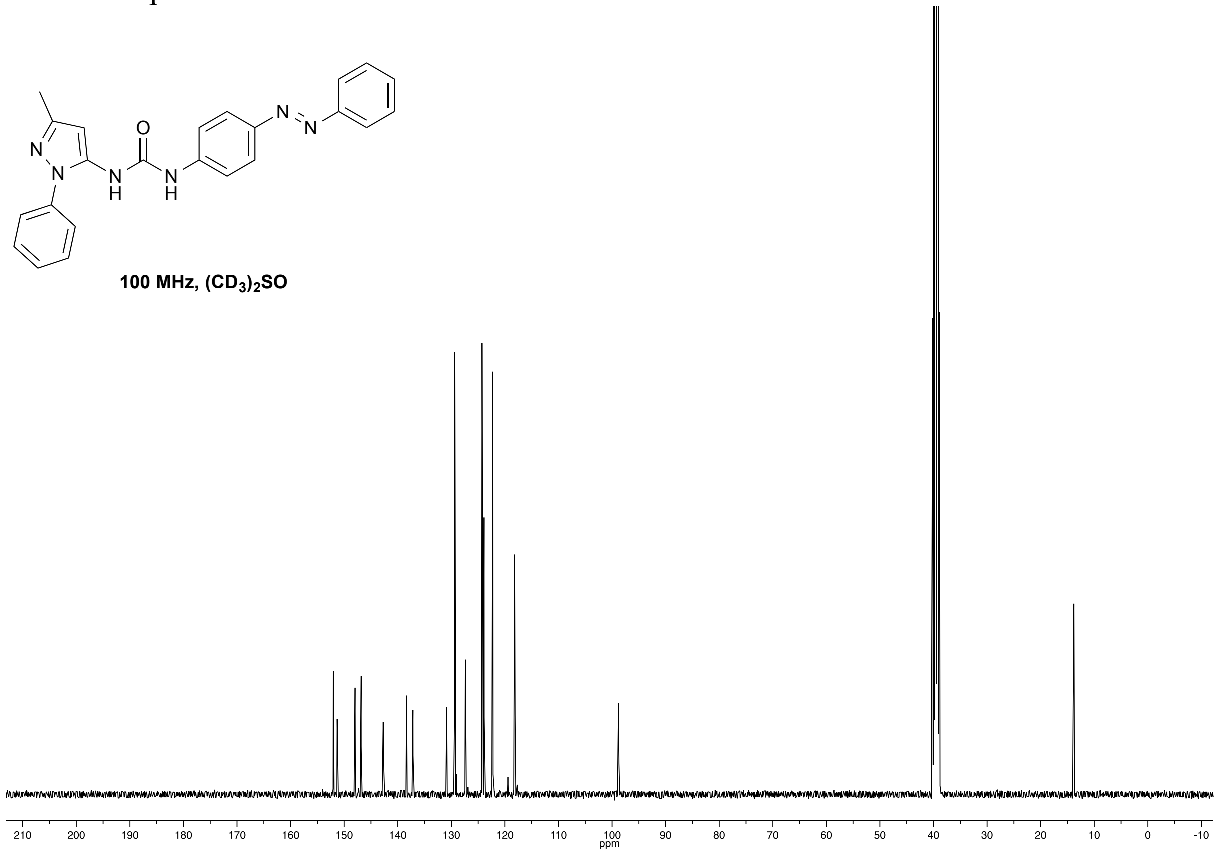

^{1}H NMR spectrum of **LOGO2**

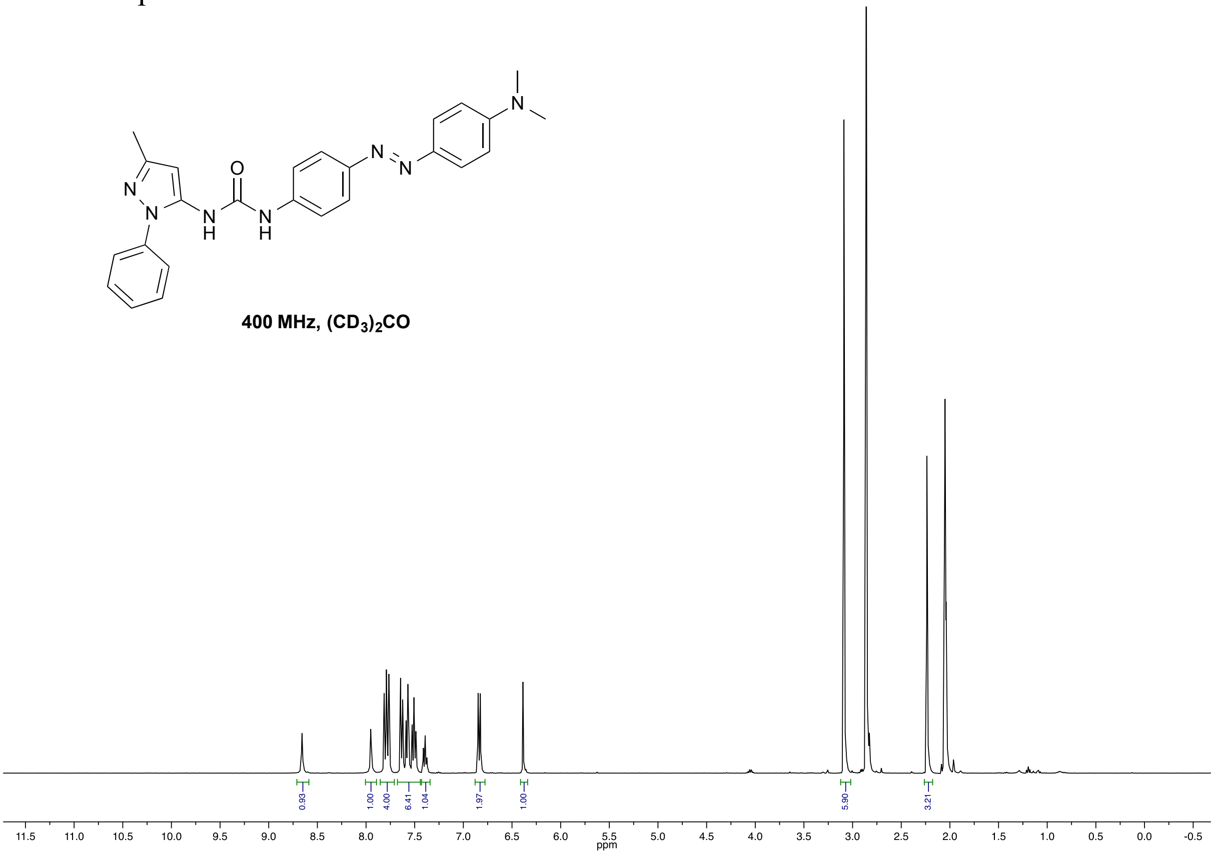

^{13}C NMR spectrum of **LOGO2**

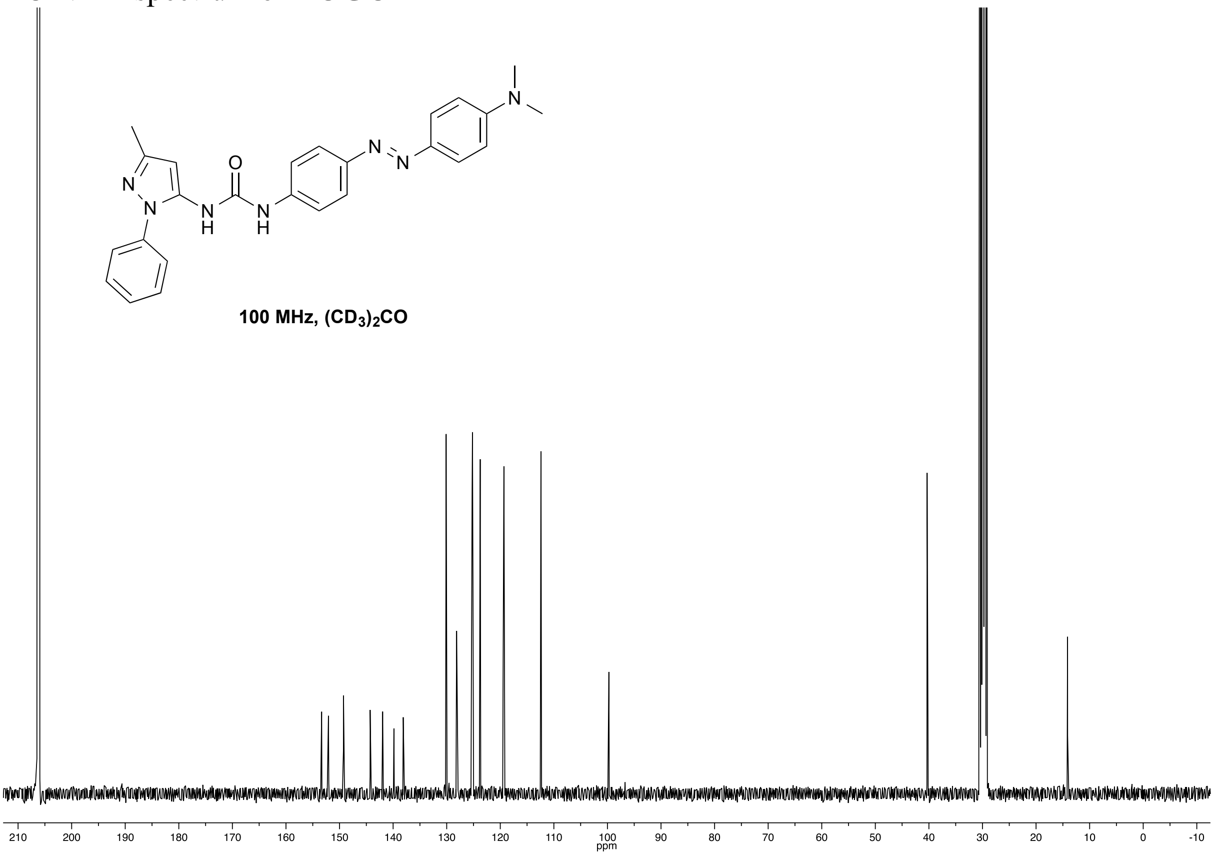

^{1}H NMR spectrum of **LOGO3**

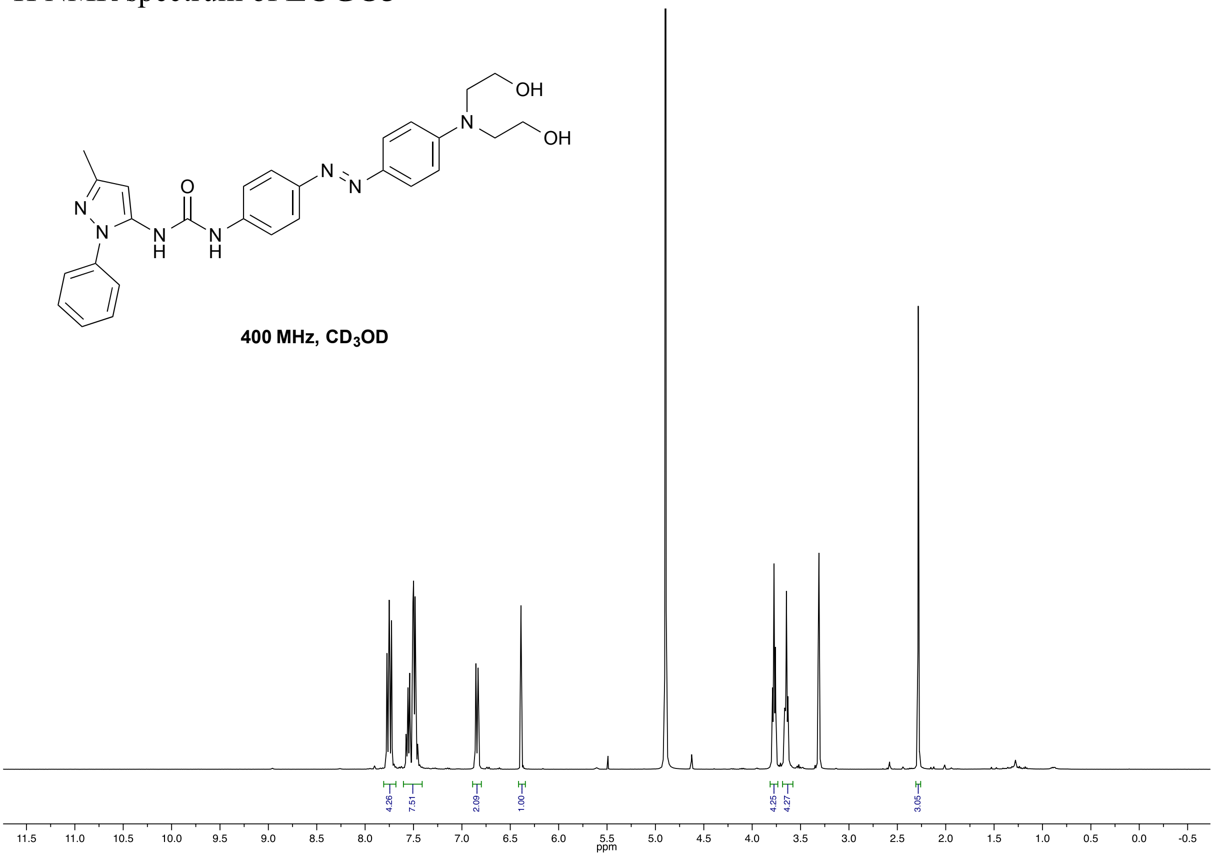

^{13}C NMR spectrum of **LOGO3**

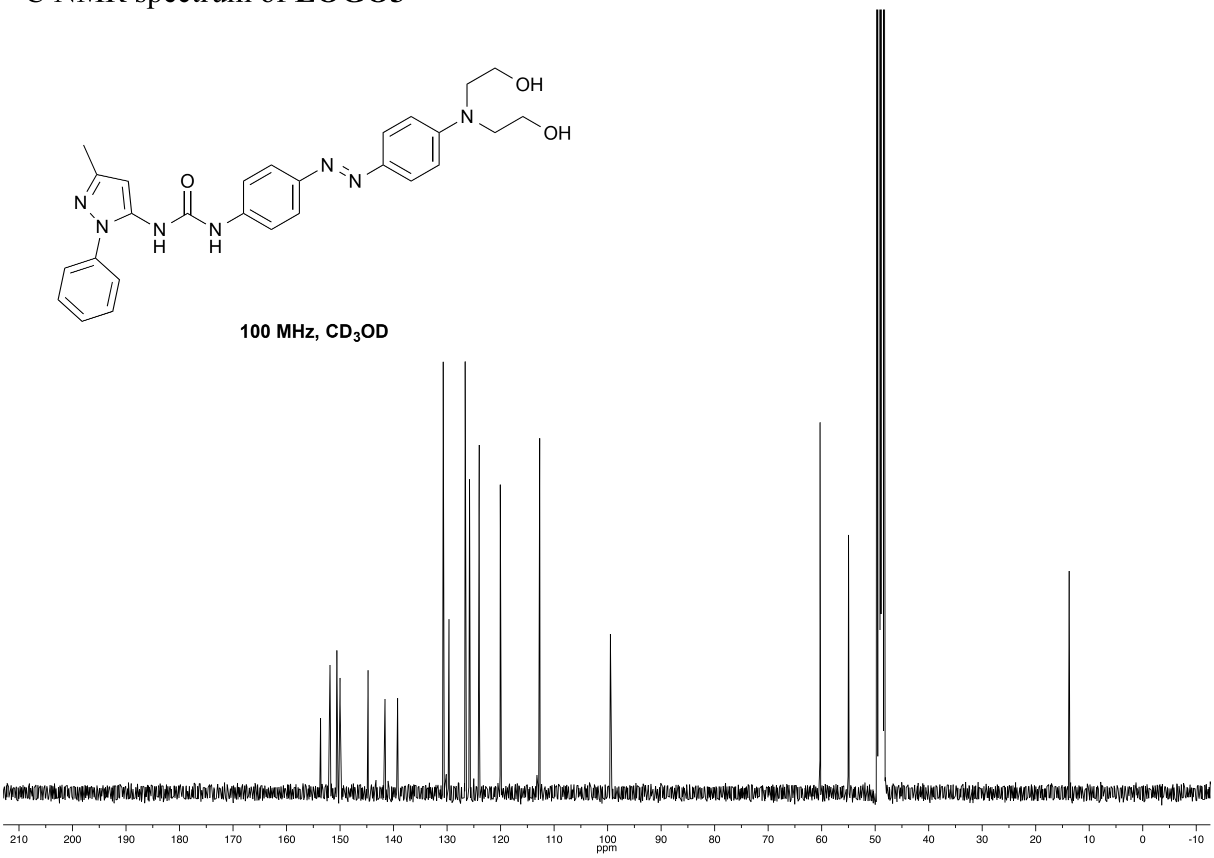

^{1}H NMR spectrum of **LOGO4**

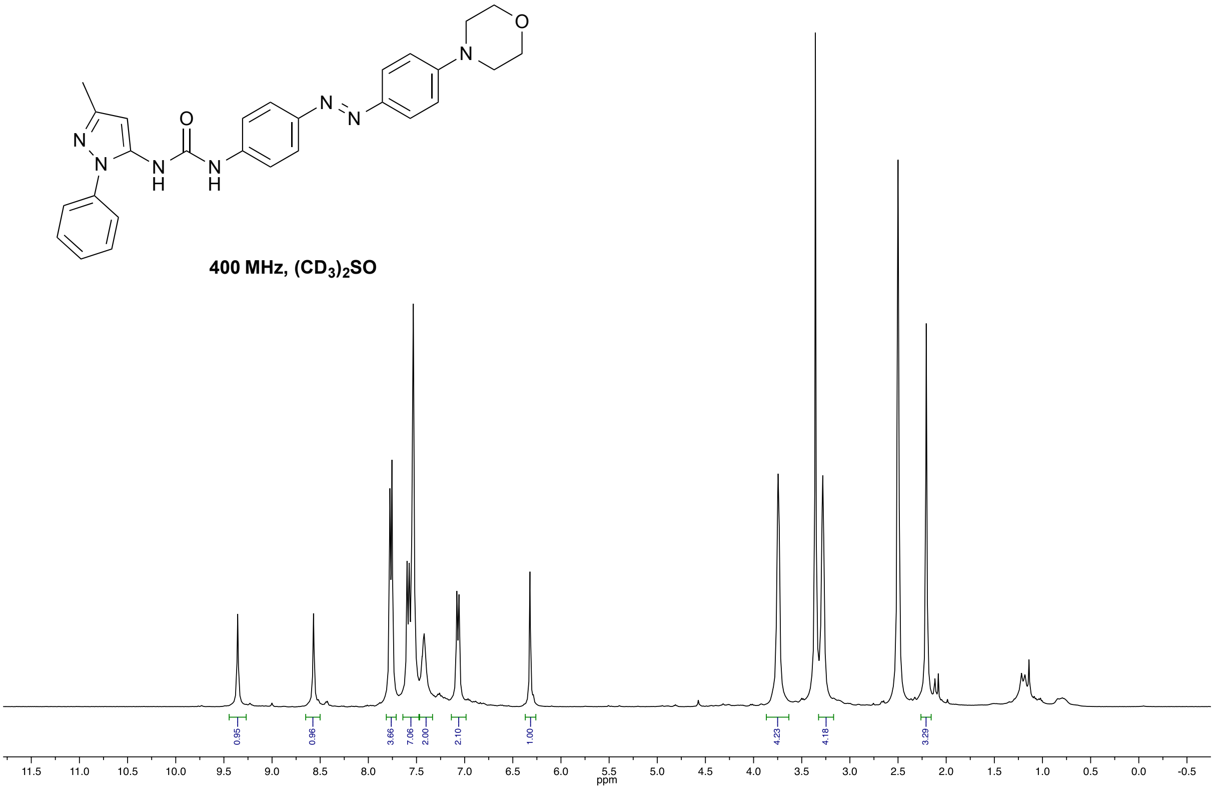

^{13}C NMR spectrum of **LOGO4**

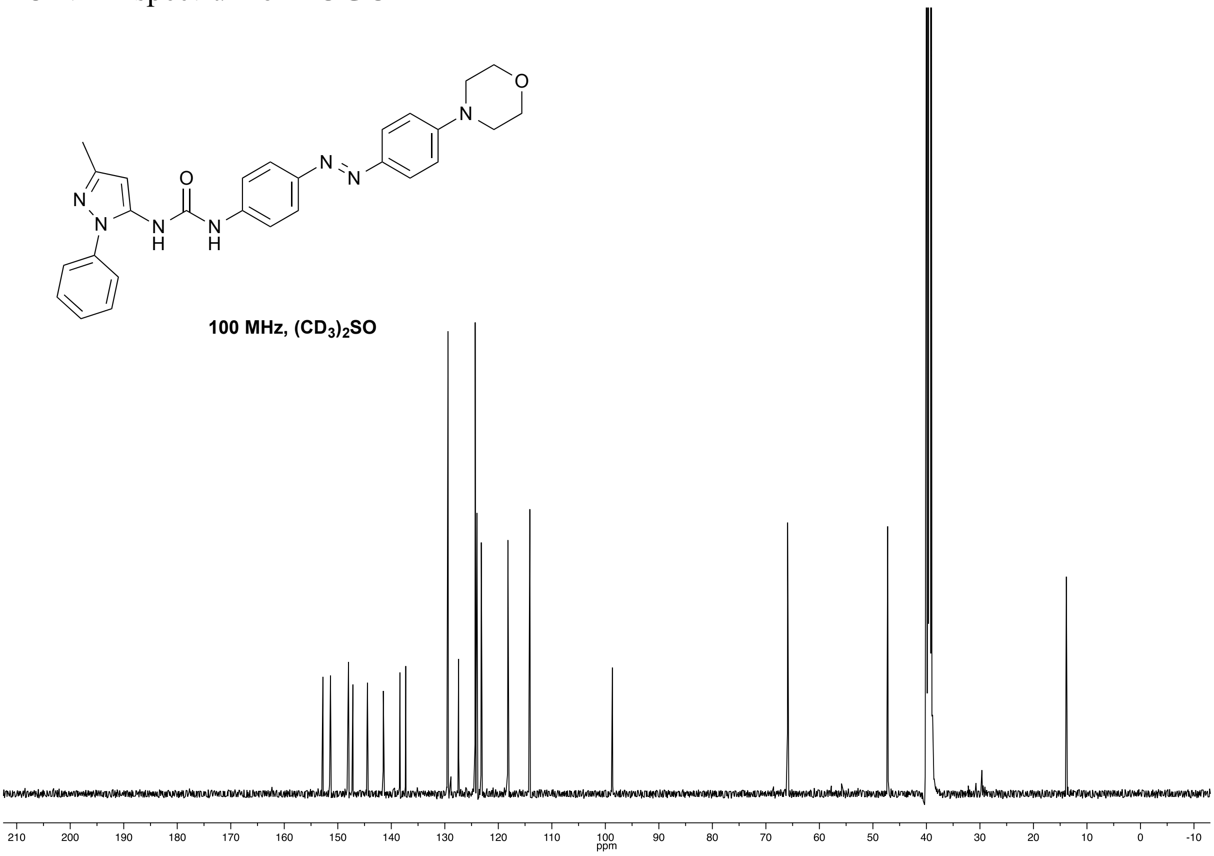

^{1}H NMR spectrum of compound **3**

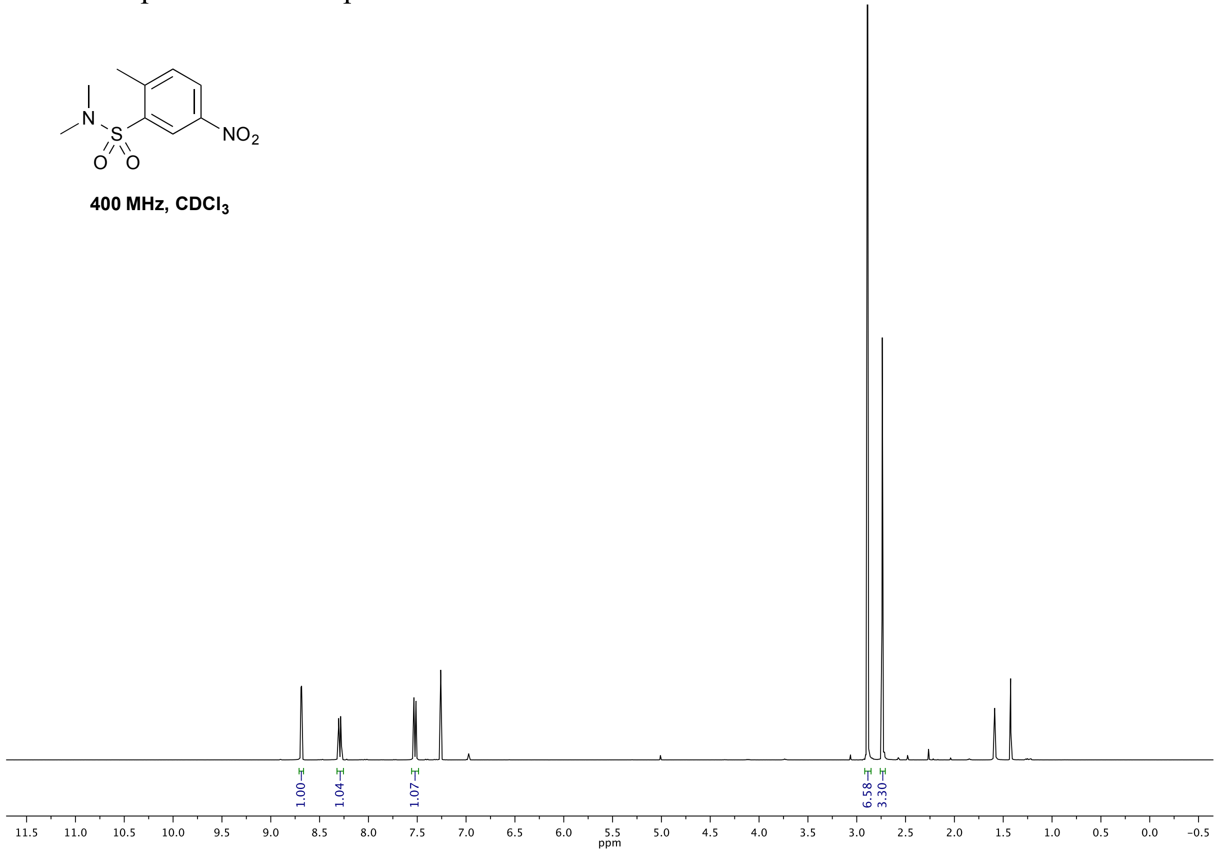

^{13}C NMR spectrum of compound **3**

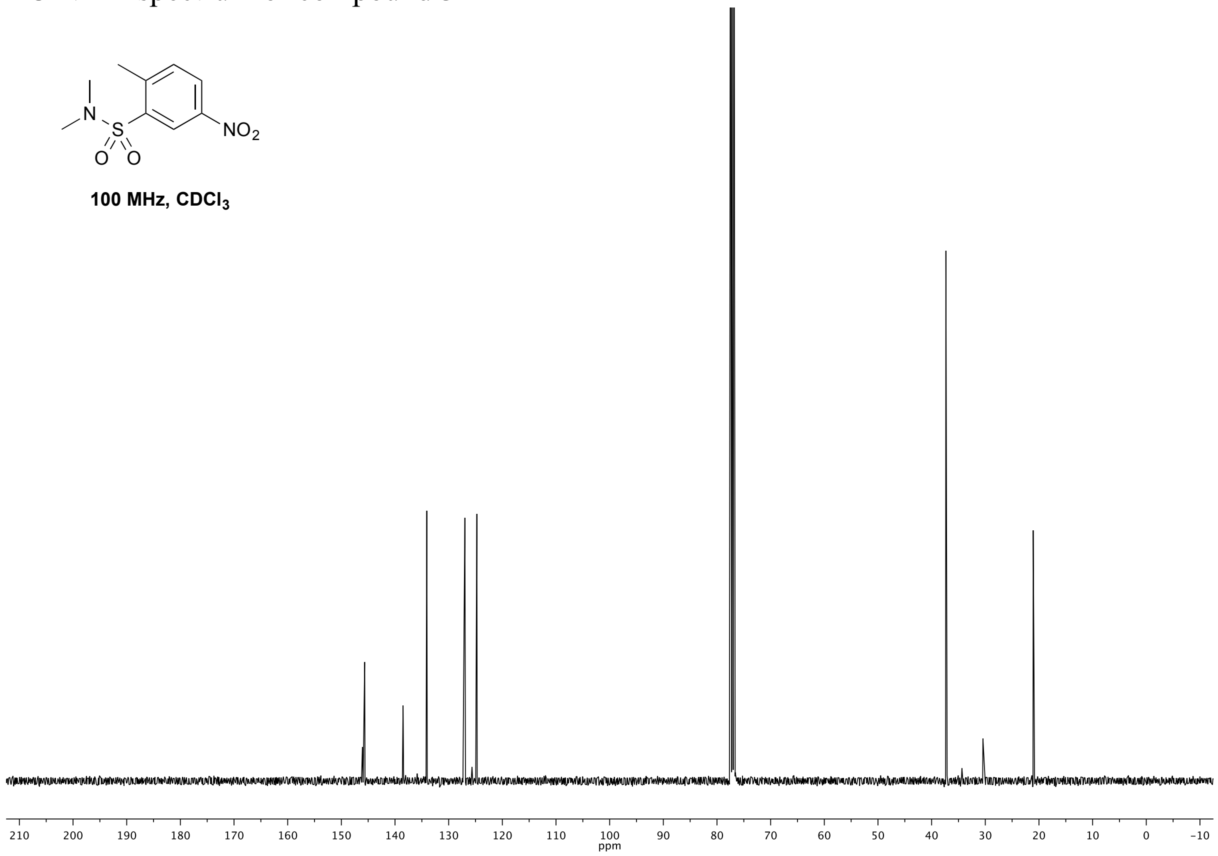

^{1}H NMR spectrum of compound **4**

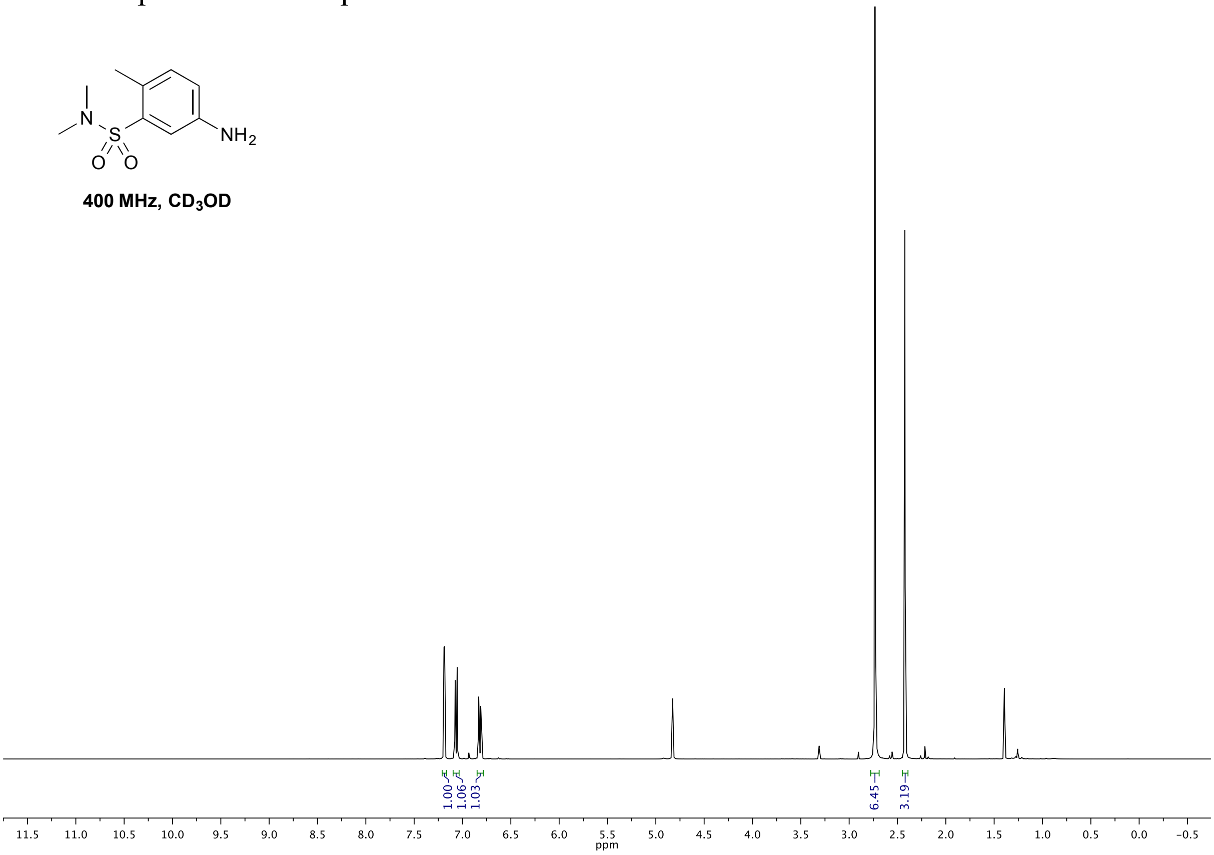

^{13}C NMR spectrum of compound **4**

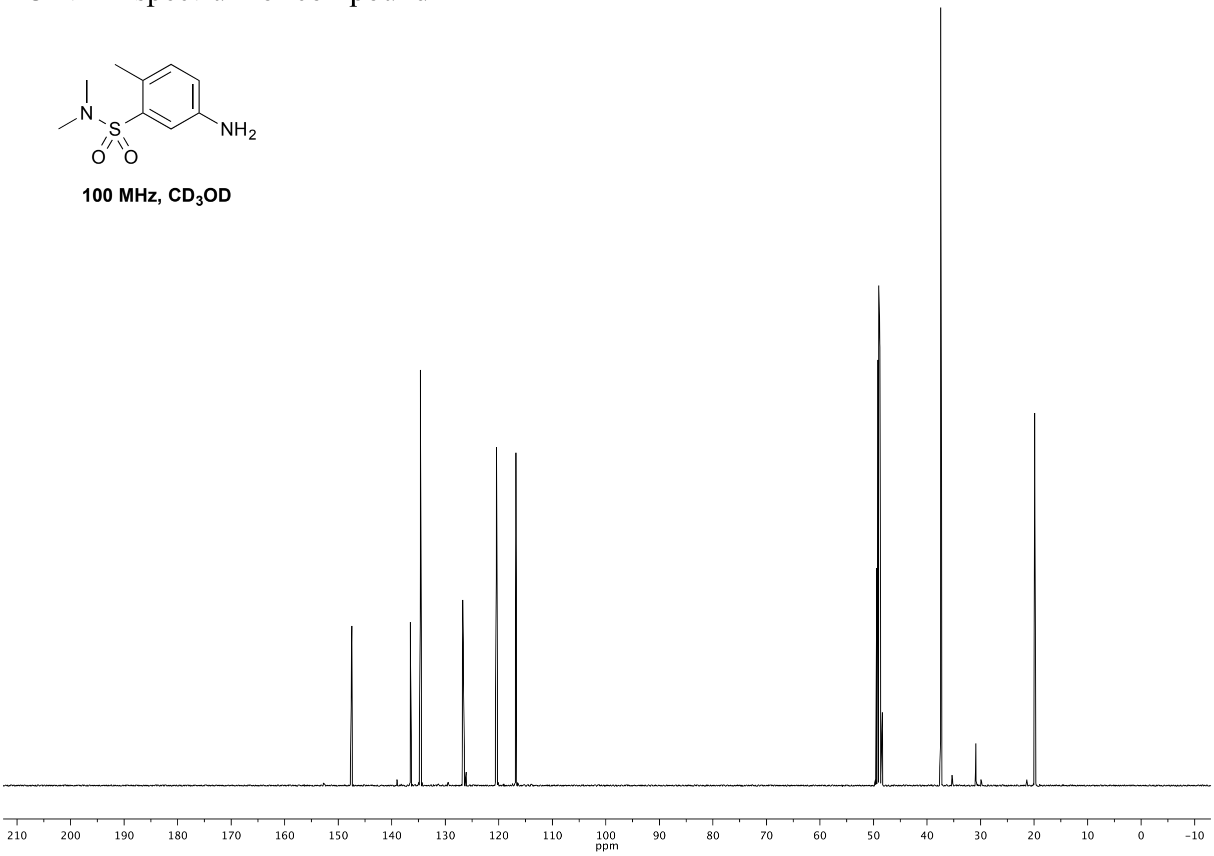

^{1}H NMR spectrum of compound **6**

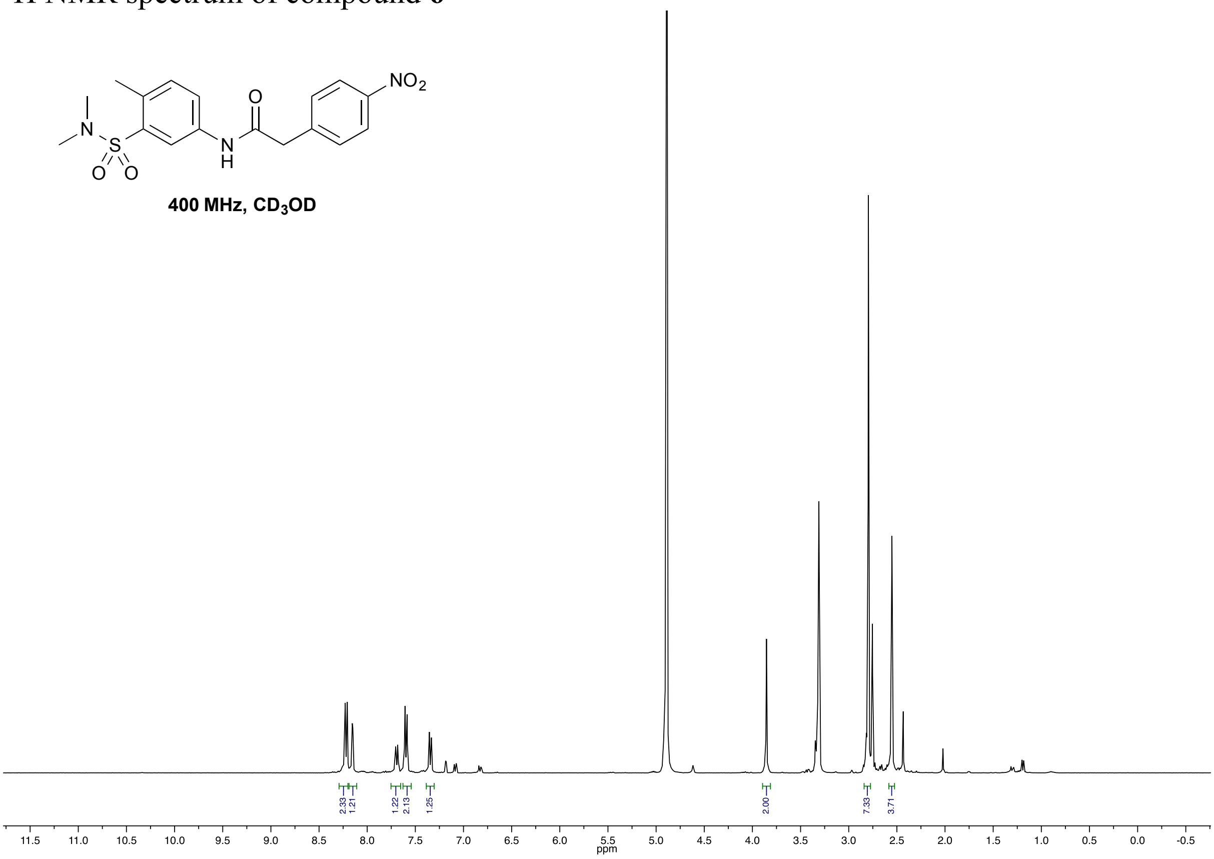

^{13}C NMR spectrum of compound **6**

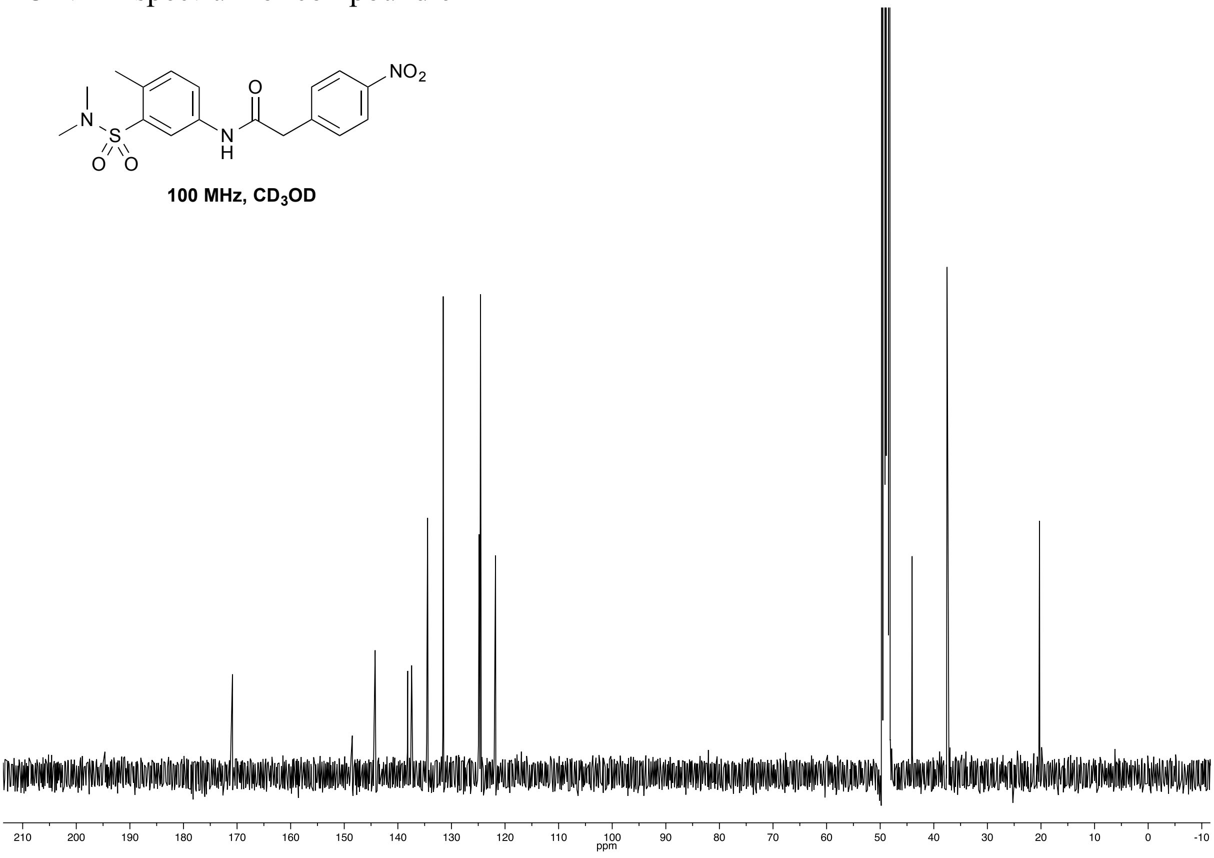

^{1}H NMR spectrum of compound **7**

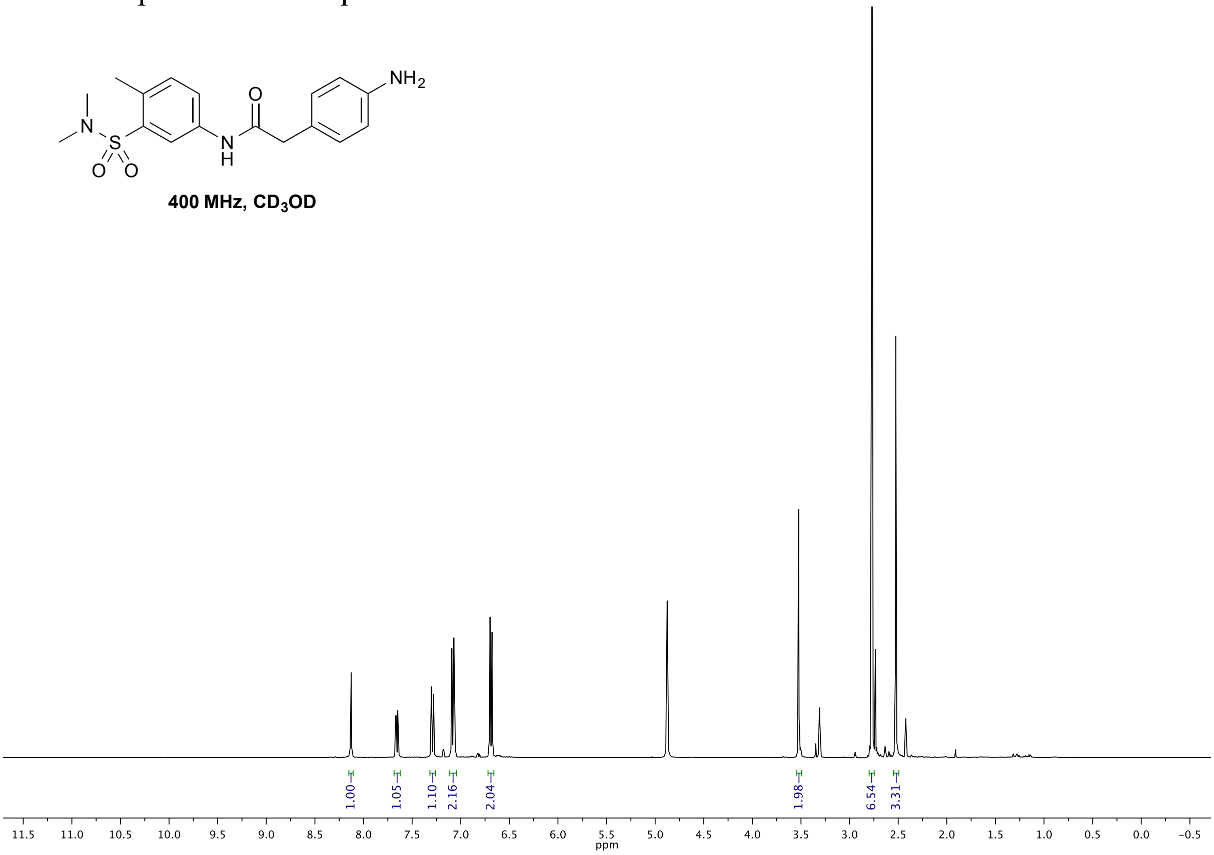

^{13}C NMR spectrum of compound **7**

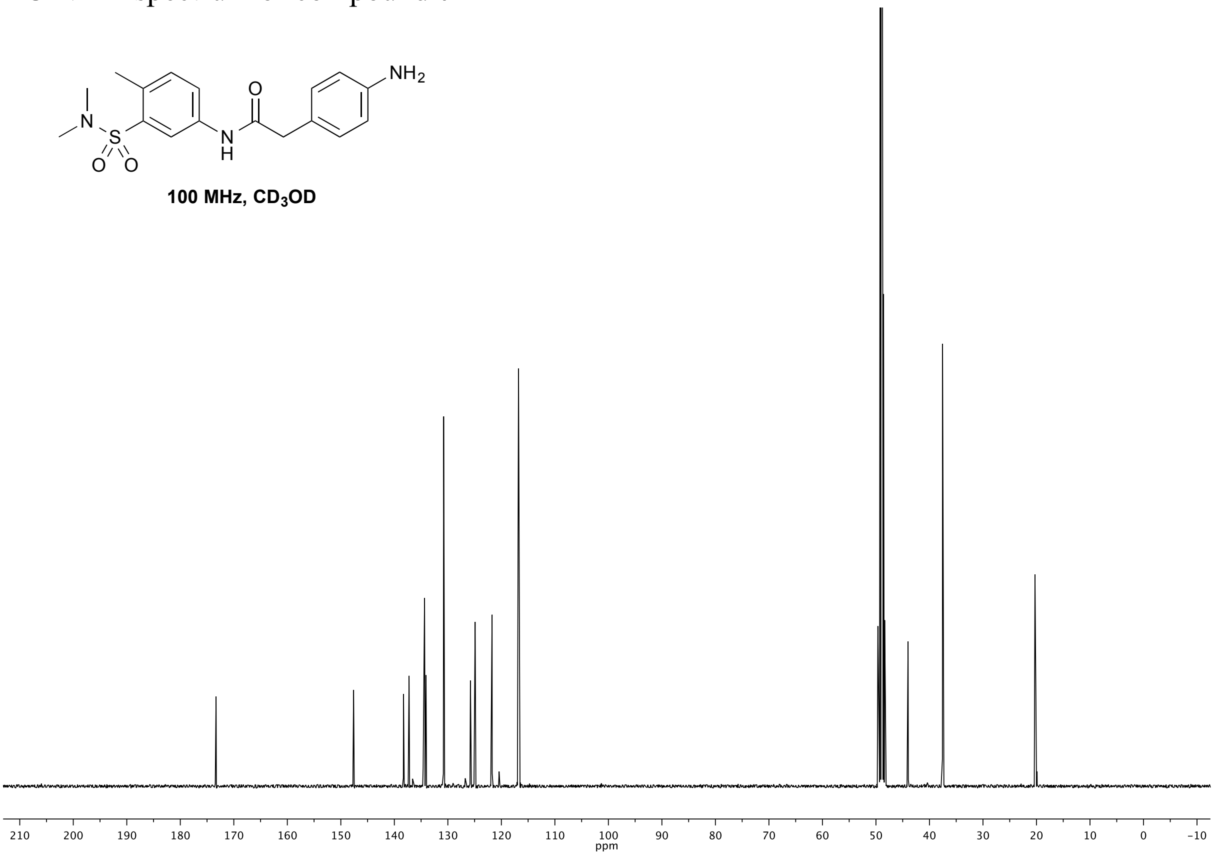

^{1}H NMR spectrum of ***trans*-LOGO5**

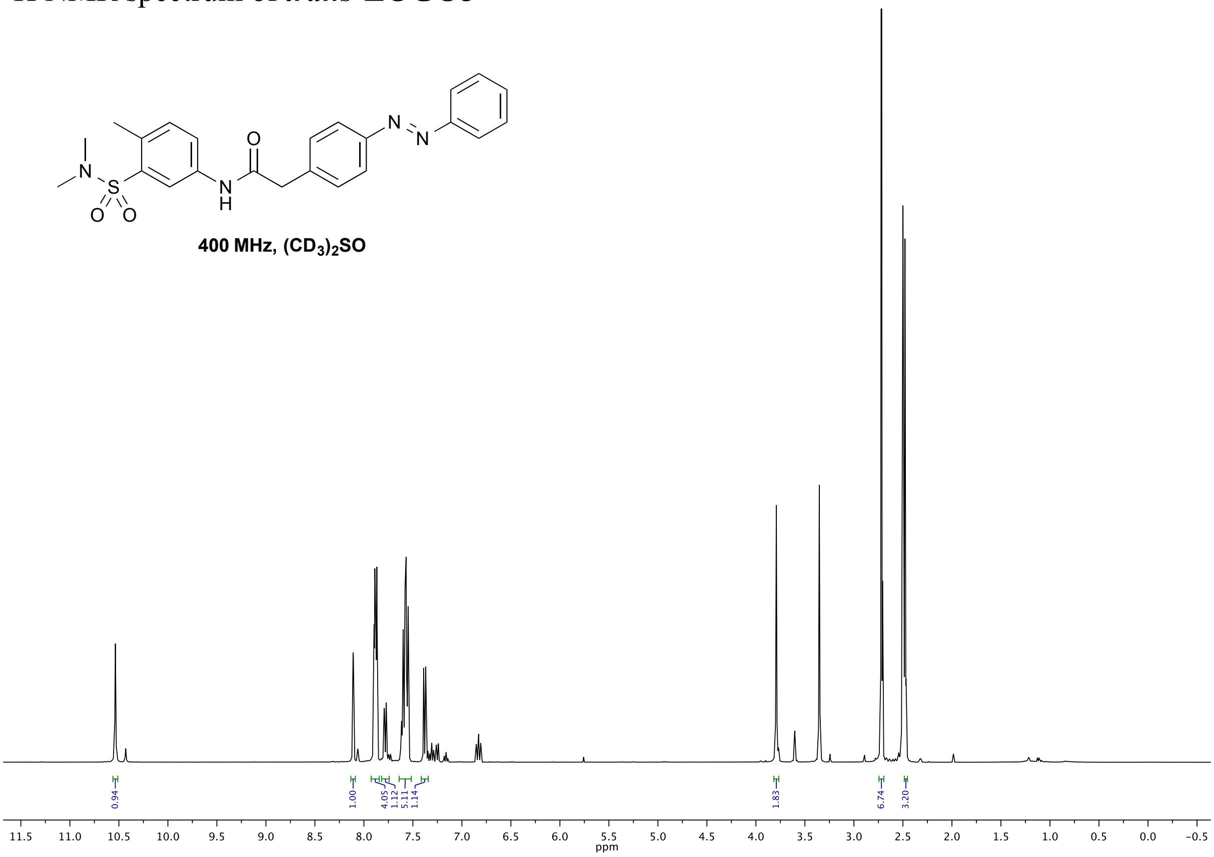

^{13}C NMR spectrum of ***trans*-LOGO5**

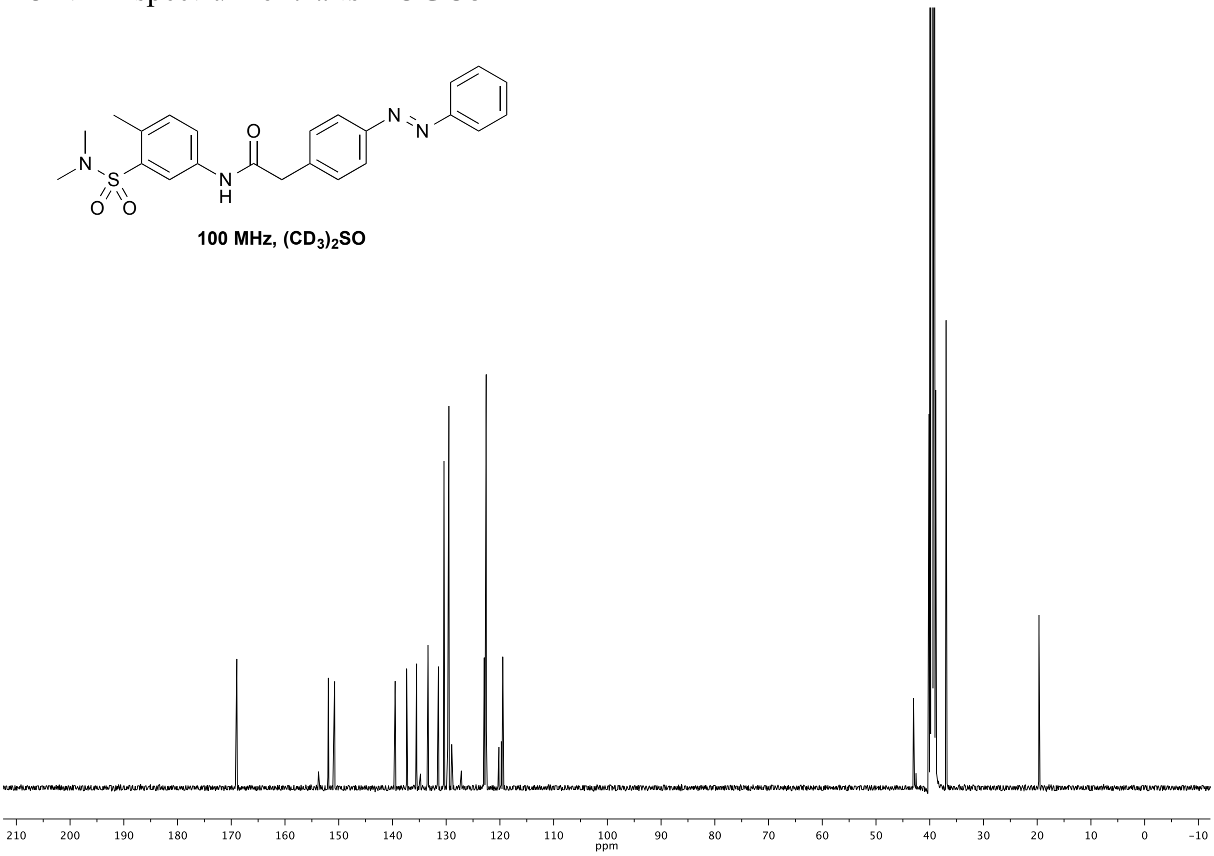

^{1}H NMR spectrum of ***cis*-LOGO5**

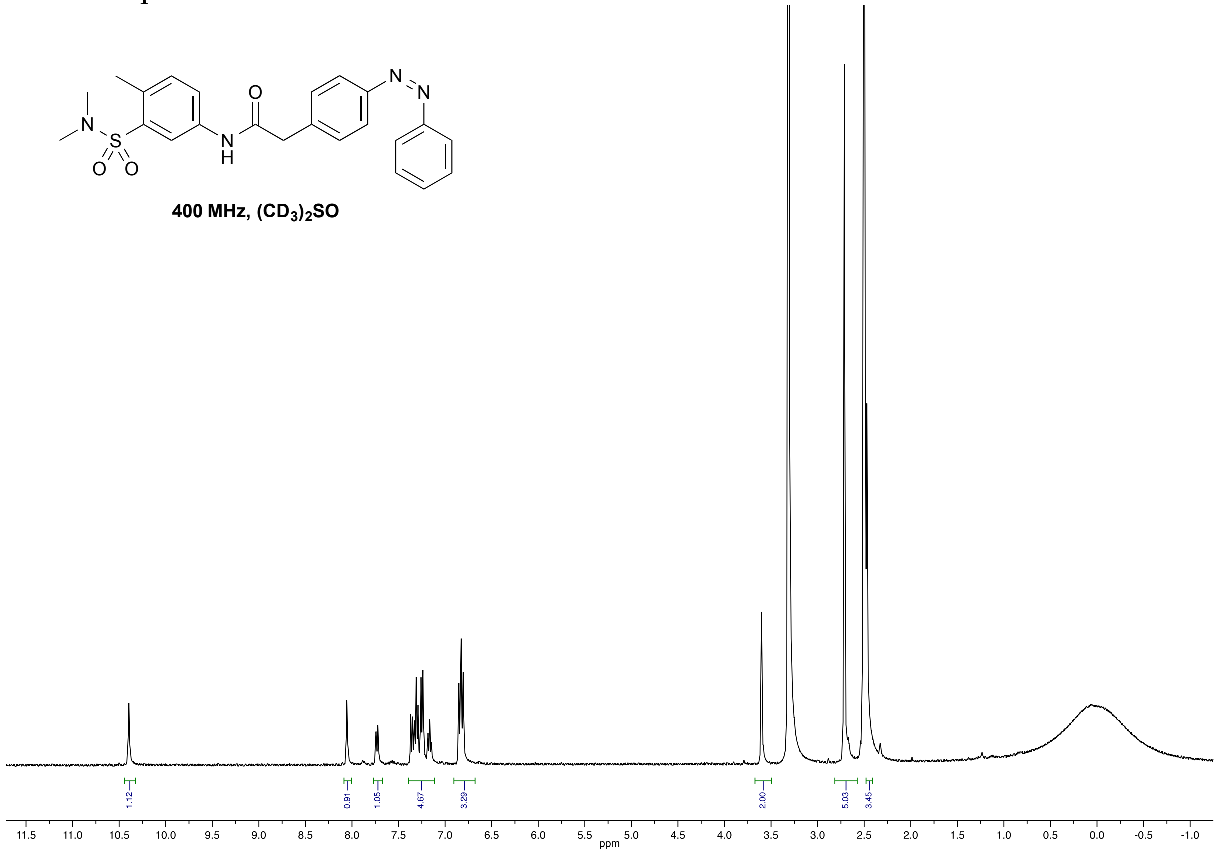

^{13}C NMR spectrum of ***cis*-LOGO5**

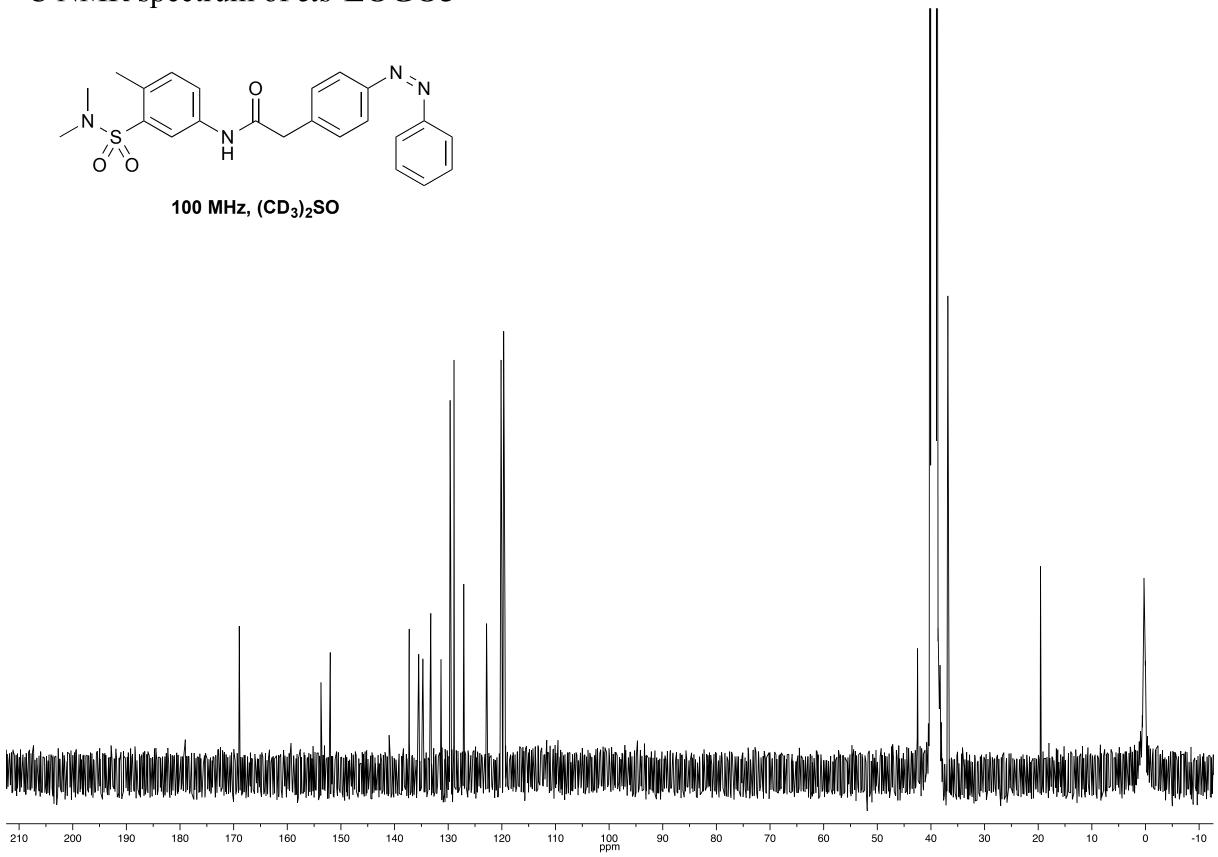

^{1}H NMR spectrum of **LOGO6**

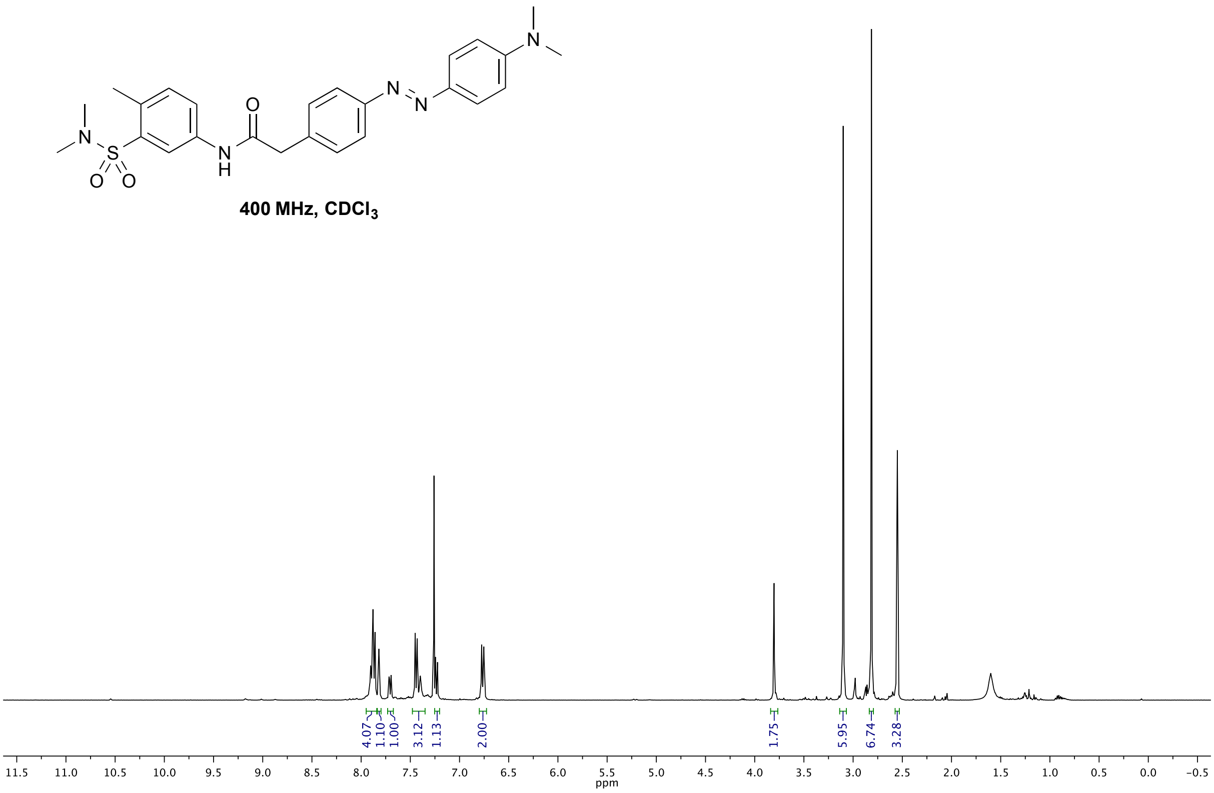

^{13}C NMR spectrum of **LOGO6**

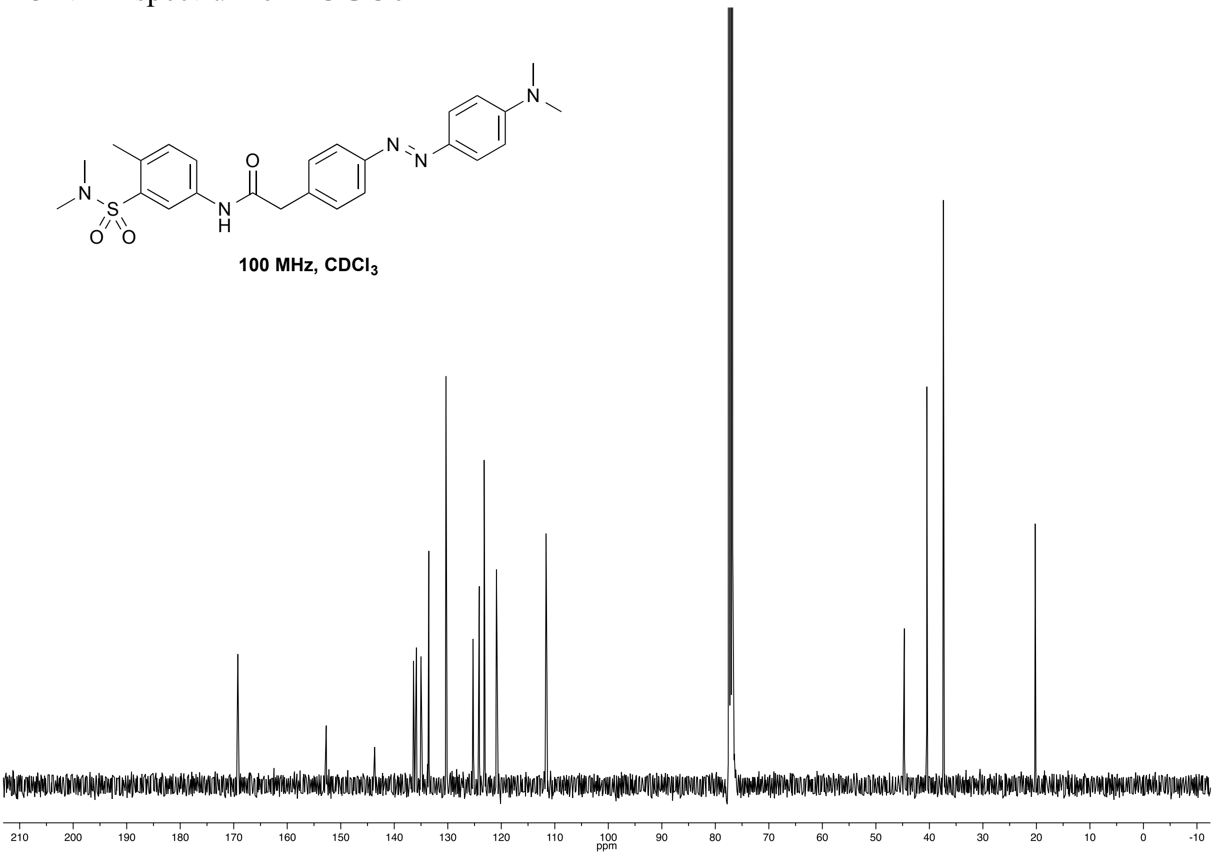

^{1}H NMR spectrum of **VU0259369**

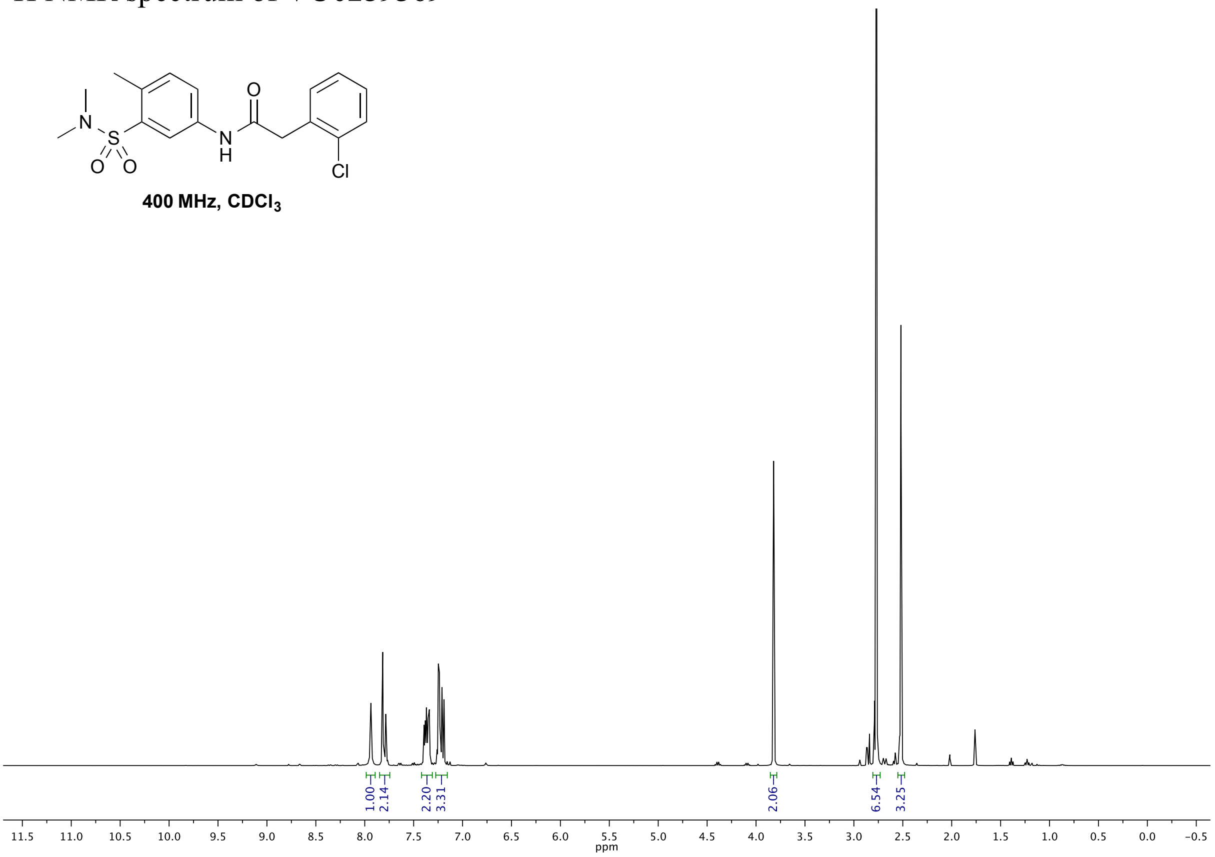

^{13}C NMR spectrum of **VU0259369**

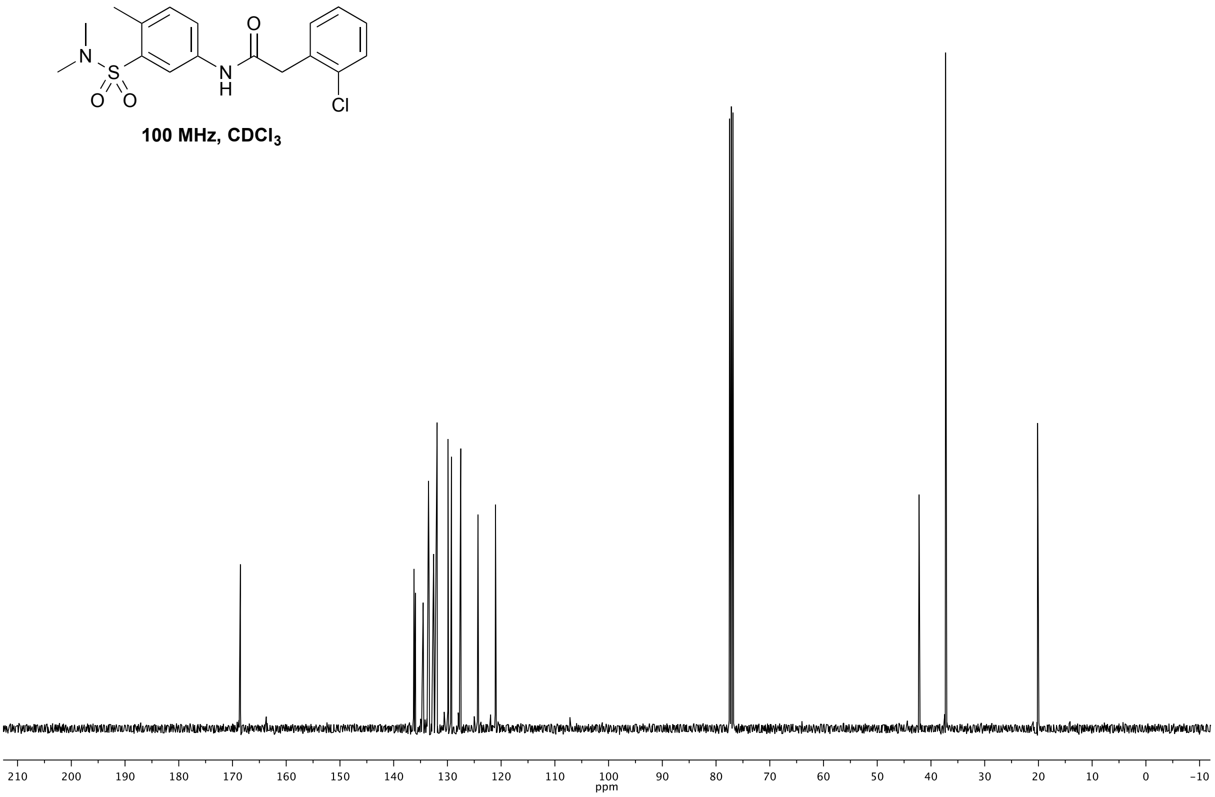

^{1}H NMR spectrum of **ML297**

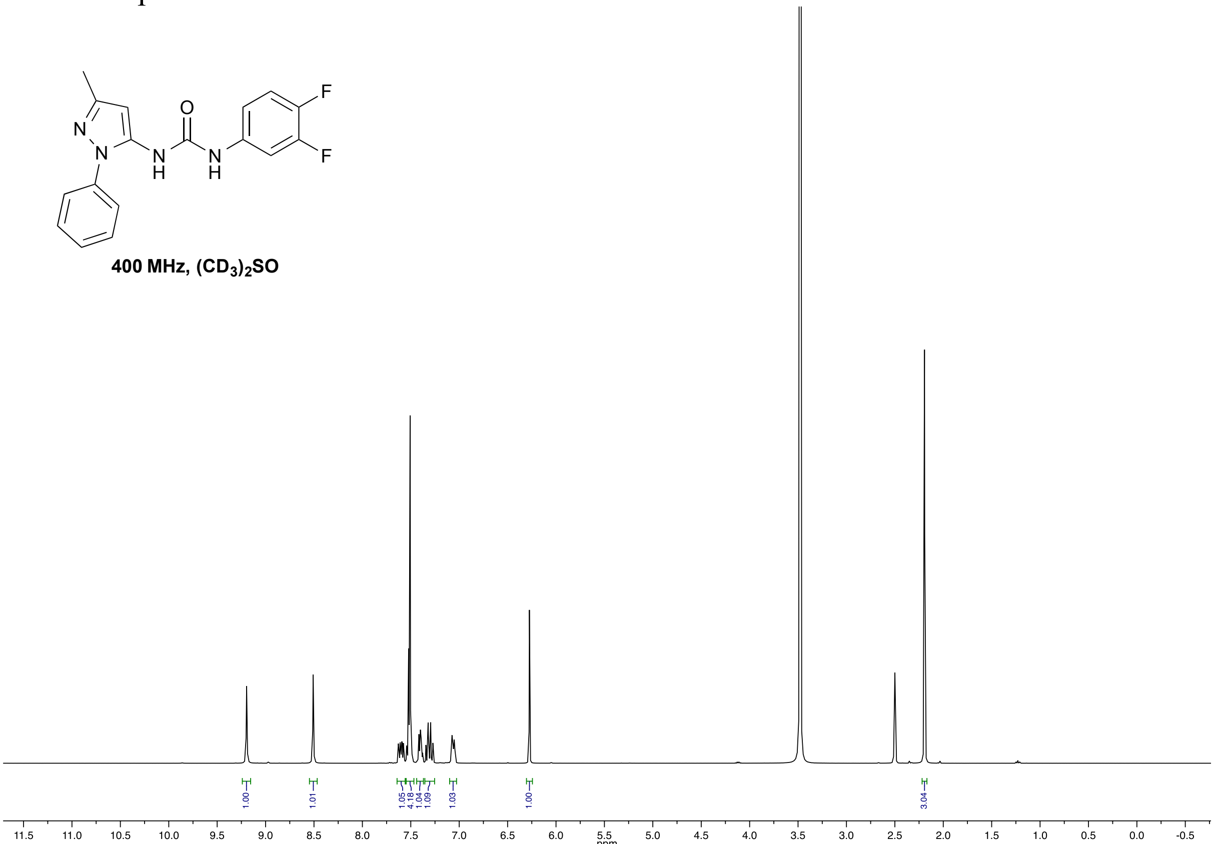

^{13}C NMR spectrum of **ML297**

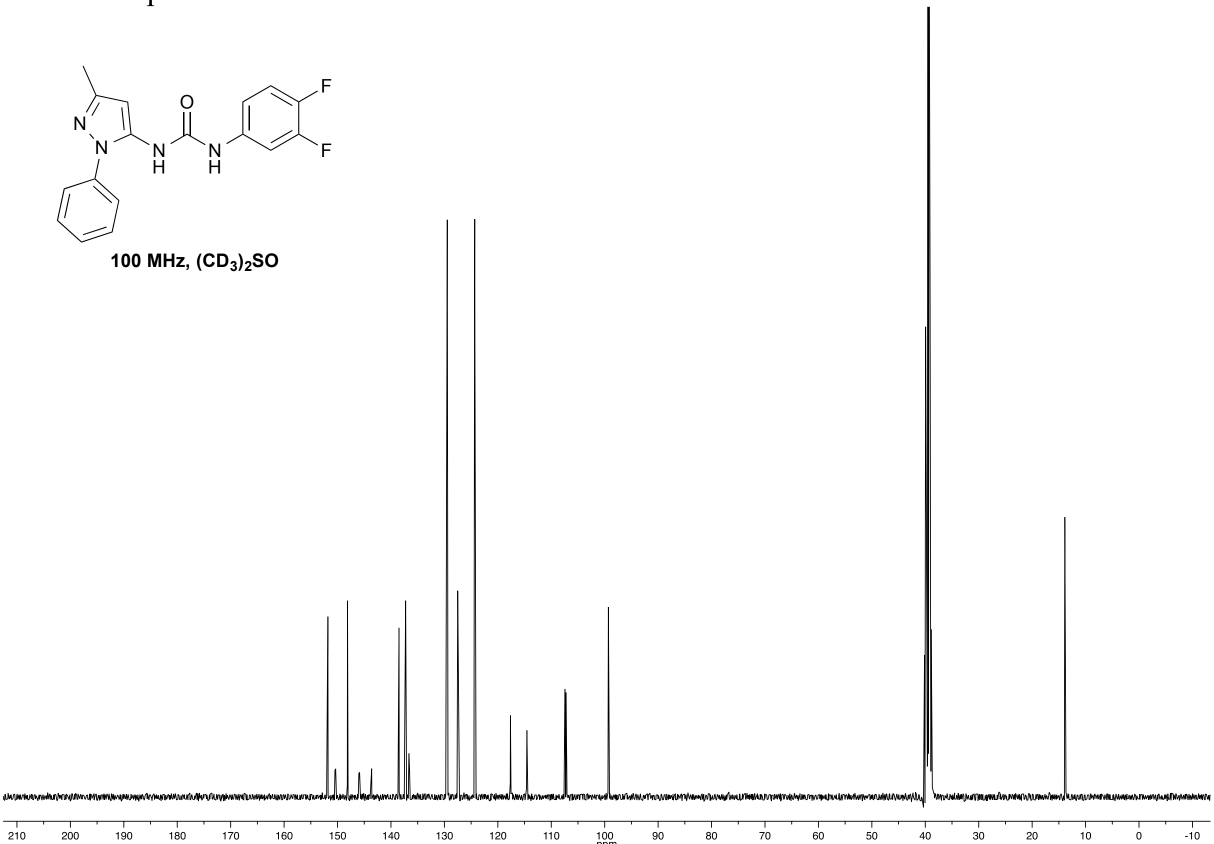

Supplement: SC-007-C5SC04084A-s001 [file SC-007-C5SC04084A-s001.pdf]
